# Whole-exome sequencing reveals a comprehensive germline mutation landscape and identifies twelve novel predisposition genes in Chinese prostate cancer patients

**DOI:** 10.1371/journal.pgen.1010373

**Published:** 2022-09-12

**Authors:** Yonghao Liang, Peter Ka-Fung Chiu, Yao Zhu, Christine Yim-Ping Wong, Qing Xiong, Lin Wang, Jeremy Yuen-Chun Teoh, Qin Cao, Yu Wei, Ding-Wei Ye, Stephen Kwok-Wing Tsui, Chi-Fai Ng

**Affiliations:** 1 School of Biomedical Sciences, The Chinese University of Hong Kong, Hong Kong, China; 2 S.H. Ho Urology Centre, Department of Surgery, Prince of Wales Hospital, The Chinese University of Hong Kong, Hong Kong, China; 3 Department of Urology, Fudan University Shanghai Cancer Center, Shanghai, China; 4 Department of Oncology, Shanghai Medical College, Fudan University, Shanghai, China; 5 Hong Kong Bioinformatics Centre, The Chinese University of Hong Kong, Hong Kong, China; Dana-Farber Cancer Institute, UNITED STATES

## Abstract

Prostate cancer is the most inheritable cancer with approximately 42% of disease risk attributed to inherited factors by studies of twins, indicating the importance of additional genetic screening to identify predisposition variants. However, only DNA damage repair (DDR) genes have been investigated thoroughly in prostate cancer. To determine the comprehensive germline mutation landscape in Chinese prostate cancer patients, we performed whole exome sequencing in 100 Han Chinese patients with prostate cancer in Hong Kong and identified deleterious germline mutations. A total of 36 deleterious germline variants in 25 genes were identified in 29% patients. Variants were found in eight pathways, including DNA methylation, DDR, and tyrosine-protein kinase. These findings were validated in an independent Chinese cohort of 167 patients with prostate cancer in Shanghai. Seven common deleterious-variant-containing genes were found in discovery cohort (7/25, 28%) and validation cohort (7/28, 25%) with three genes not described before (*LDLR*, *MYH7* and *SUGCT*) and four genes previously reported (*FANCI*, *ITGA6*, *PABPC1* and *RAD54B*). When comparing with that of a cohort of East Asian healthy individuals, 12 non-DDR novel potential predisposition genes (*ADGRG1*, *CHD4*, *DNMT3A*, *ERBB3*, *GRHL1*, *HMBS*, *LDLR*, *MYH7*, *MYO6*, *NT5C2*, *NUP98* and *SUGCT*) were identified using the discovery and validation cohorts, which have not been previously reported in prostate cancer patients in all ethnic groups. Taken together, this study reveals a comprehensive germline mutation landscape in Chinese prostate cancer patients and discovers 12 novel non-DDR predisposition genes to lay the groundwork for the optimization of genetic screening.

## Author summary

Prostate cancer is the most inheritable cancer with about 42% of disease risk attributed to inherited factors, indicating the importance of additional genetic screening to identify predisposition variants. However, only DNA damage repair (DDR) genes have been studied thoroughly in prostate cancer. To determine the comprehensive germline mutation landscape in Chinese prostate cancer patients, we performed whole exome sequencing in 100 Han Chinese patients with prostate cancer in Hong Kong and identified deleterious germline mutations. A total of 36 deleterious germline variants in 25 genes were identified in 29% patients. Variants were found in eight pathways, including DNA methylation, DDR, and tyrosine-protein kinase. These findings were validated in an independent Chinese cohort of 167 patients with prostate cancer in Shanghai. Seven common deleterious-variant-containing genes were found in discovery cohort and validation cohort with three genes not described before (*LDLR*, *MYH7* and *SUGCT*) and four genes previously reported. When comparing with that of a cohort of East Asian healthy individuals, 12 non-DDR novel potential predisposition genes were identified using the discovery and validation cohorts, which have not been previously reported in prostate cancer patients in all ethnic groups.

## Introduction

Inherited genetic factors notably contribute to breast cancer, colorectal cancer, and prostate cancer susceptibility. In prostate cancer, approximately 42% of disease risk is attributed to inherited factors, which makes it one of the most inheritable cancers [1]. Whole exome sequencing (WES) has been shown to be one of the most cost-effective methods to investigate germline mutations associated with inherited human cancers. According to the National Comprehensive Cancer Network 2020 clinical practice guidelines on prostate cancer, germline mutation testing is recommended in patients with high risk, very high risk, regional metastases, and distant metastases [2]. In addition, genetic testing in prostate cancer is also used to select the optimal therapeutic strategy. Germline mutations in several DNA damage repair (DDR) genes, such as *BRCA1* and *BRCA2*, which have been verified as susceptibility factors for prostate cancer, are also predictors of the response to poly(ADP-ribose)polymerase inhibitor therapy or platinum-containing chemotherapy [3–5]. Additionally, germline mutations in other DDR genes such as *MLH1*, *MSH2*, *MSH6*, and *PMS2* have been reported to predict response to immunotherapy in advanced prostate cancer patients [6,7]. Finally, previous studies have demonstrated that patients with metastatic prostate cancer and harboring germline mutations of specific variants in DDR would have variable responses to systemic hormone therapy depending on such variants [8–11].

With the development and reduction of the cost of WES, a growing body of studies have used WES to investigate the germline mutation landscape and its association with clinical characteristics in prostate cancer patients. A multi-institutional study involving 150 metastatic castration-resistant prostate cancer (mCRPC) patients identified germline mutation variants enriched in several biological pathways, including androgen receptor (AR) signaling, phosphatidylinositol-4,5-bisphosphate 3-kinase (PI3K), Wnt, cell cycle, and DNA repair pathways [12]. Another study investigating the mutations in a 20 DDR genes reported that 11.8% of metastatic prostate cancer patients carried germline mutations and that the incidence of germline mutations in men with localized prostate cancer was significantly lower [13]. Using a clinician-selected multigene panel, a cross-sectional study of 3,607 men with a personal record of prostate cancer revealed that approximately 17% of patients carried a pathogenic germline variant [14]. In 2019, a study using a panel composed of 18 DDR genes and comprising 316 Chinese prostate cancer patients reported that 9.8% of these patients carried pathogenic germline mutations [15]. More recently, in a cohort of 246 Chinese patients, Wu et al.[16] found that 31% of prostate cancer patients harbored pathogenic germline mutations in a panel of selected 276 DDR genes [16].

Although in recent years there have been substantial advances in dissecting the germline mutation landscape in prostate cancer, no consensus has been achieved regarding the most adequate selection of genes for assessment. Consequently, considerable variations in the results of these studies are expected. In addition, most of the studies conducted up to date thoroughly investigated only DDR genes, hindering obtaining a comprehensive overview of the germline mutation landscape in other genetic pathways involved in the pathogenesis of prostate cancer. Moreover, in the most commonly used gene panels, only DDR genes involved in homologous recombination and mismatch repair pathways were included, and many other important DNA repair pathways such as base excision repair, nucleotide excision repair, direct damage reversal/repair were omitted [17]. Furthermore, most of the previous studies have investigated the germline mutation landscape in the Caucasian population, and populations of other ethnicities such as the Chinese population have not been thoroughly investigated. Given that genetic effects are ethnic-specific, it is of great necessity to dissect the germline mutation landscape in Chinese prostate cancer patients [18].

To explore the genetic basis of prostate cancer in Chinese patients and to identify candidate predisposition genes, in this study we performed WES of germline DNA from 100 prostate cancer patients in Hong Kong and compared our results with East Asian individuals from the GnomAD cohort. Our results were then validated in another independent Chinese prostate cancer cohort in Shanghai. Moreover, gene function enrichment analysis and correlation analysis with clinical characteristics were conducted. The results of this study provide a comprehensive germline mutation profile of Chinese prostate cancer.

## Material and methods

### Ethics statement

The study in Hong Kong was approved by the Joint Chinese University of Hong Kong–New Territories East Cluster Clinical Research Ethics Committee (The Joint CUHK-NTEC CREC) (CREC 2015.444). The study in Shanghai was approved by Fudan University Shanghai Cancer Center, Shanghai, China (050432-4-1911D). All patients had signed informed consent for the studies.

### Study design

We undertook a study of germline mutations among patients with prostate cancer using WES. We then compared our results with 9,197 East Asian individuals from the GnomAD v2.1.1 cohort [19] and validated our results in an independent Chinese cohort of 167 patients with prostate cancer. Finally, germline genotypes were correlated with patient clinical characteristics.

### Enrollment of patients

From 2008 to 2016, a total of 100 Chinese men with known prostate cancer in Hong Kong were included. The independent validation cohort consisted of 167 ethnically Chinese men with prostate cancer in Shanghai. Patients would be recruited into the studies if they contained either one of the following inclusion criteria: (1) age of diagnosis less than 60 years old, or (2) Gleason score greater than 7, or (3) metastatic disease at presentation. Clinical and demographic information were prospectively collected through the electronic medical records. Personal and family history of cancer were obtained in person by a standardized questionnaire.

### DNA extraction and WES

Genomic DNA was extracted from patients’ peripheral blood mononuclear cells by using a QIAamp DNA Blood Mini Kit. Afterwards, genomic DNA was randomly fragmented by Covaris technology into 150bp and 250bp and size-selected DNA fragments were purified and hybridized to the exome array for enrichment. High-throughput sequencing was performed in DNBseq sequencing platforms by BGI. By applying the Burrows-Wheeler Aligner, the paired-end reads were aligned to the human reference hg19 [20]. After removing the PCR duplicates by Picard, variant calling was performed by the Genome Analysis Toolkit [21].

### Data analysis of germline mutations

**Fig 1:**
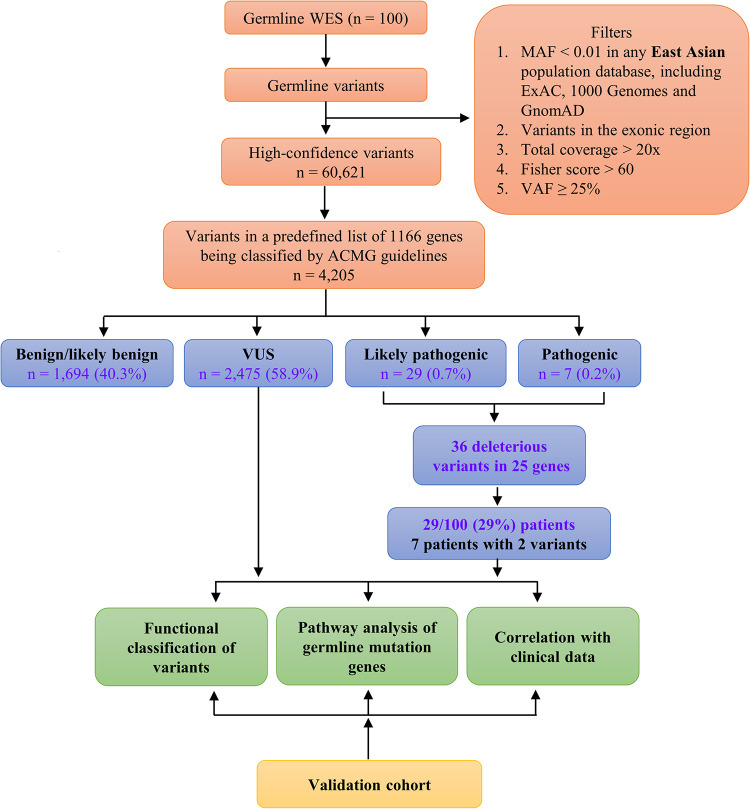
Overview of the data analysis strategy to identify candidate prostate cancer susceptibility genes. A total of 100 Hong Kong patients with prostate cancer were included. The patient characteristics are summarized in Table 1. Germline samples were whole exome–sequenced and aligned to human genome assembly hg19 before variant calling and annotations. All germline variants were identified and filtered by (i) MAF < 0.01 in any East Asian population database including ExAC, 1000 Genomes and GnomAD v2.1., (ii) variants in the exonic region, (iii) variants with total coverage > 20x, (iv) variants with Fisher score > 60, and (v) VAF ≥ 25%. Among the 60,621 variants after filtering, 4,205 variants belonging to a predefined list of 1,166 genes were annotated as pathogenic or likely pathogenic (deleterious), variant of uncertain significance (VUS), likely benign, or benign (benign) according the ACMG guidelines. The 1,166-gene list is provided in S1 Table. We also performed functional classification of variants and correlations with clinical characteristics of patients. Our data were validated with an independent prostate cancer cohort (n = 167 patients). MAF, minor allele frequency; VAF, variant allele frequency/fraction.

The sequencing data analysis was focused on the presence of single-nucleotide variants as well as small insertions and deletions. Variants were included for further analysis when they met the following criteria: (i) Minor allele frequency (MAF) < 0.01 in any East Asian population database including ExAC, 1000 Genomes and GnomAD v2.1, (ii) Variants in the exonic regions, (iii) variants with total coverage > 20x, (iv) variants with Fisher score > 60, and (v) variant allele frequency/fraction (VAF) ≥ 25%. A comprehensive predisposition gene panel was prepared for further analysis (S1 Table). There were three criteria for selecting genes into the predefined predisposition gene panel: (i) genes associated with hereditary cancers [17,22–24], (ii) cancer-associated genes (oncogenes and tumor suppressor genes based on Catalogue of Somatic Mutations in Cancer)[25] and (iii) prostate cancer predisposition genes from previously published papers [26–28]. Variants were also kept in the list if their corresponding genes were included in predefined predisposition gene panel. The selected variants were then annotated according to the ACMG guideline [22]. Functional classification of variants, pathway analysis of deleterious germline mutation genes, and correlation with clinical data were conducted. An independent cohort of 167 patients from Shanghai were analyzed by the same pipeline and the results was compared with the finding of our cohort. The workflow of our data analysis was shown in Figs 1 and S1.

An R package called clusterProfiler was applied to conduct enrichment analysis of GO and KEGG [29–32]. A software called Weka was used to conduct the logistic regression [33]. The Search Tool for the Retrieval of Interacting Genes (STRING) database was used to construct protein–protein interaction (PPI) networks [34]. The structure model of protein was generated by the online tool SWISS-MODEL [35].

### Statistical analysis

The demographic and clinical characteristics of the Hong Kong cohort were presented by descriptive statistics. Fisher’s exact tests was used to test the difference between the two groups with respect to dichotomous variables while Wilcoxon rank-sum tests were applied to detect the differences between the two groups in terms of continuous variables. Odd ratios (ORs) and 95% confidence intervals (CIs) were used to assess the association between the presence of deleterious germline variants and clinical characteristics. Fisher’s exact tests were employed to compare the frequencies of deleterious germline variants identified in our cohort with that in the East Asian individuals from the GnomAD v2.1.1 cohort. Fisher’s exact tests, ORs and CIs were calculated by MedCalc statistical software version 19.2.6 (MedCalc Software bv, Ostend, Belgium; **https://www.medcalc.org**; 2020). For the cases like singleton observation of a pathogenic variant, 0.5 was added to all cells (a, b, c, d) during the calculation with MedCalc [36,37]. Statistical analyses were performed with PRISM software version 9.0.2 and R version 4.0.5. All statistical tests were two-sided and Bonferroni adjusted. P < 0.05 was considered statistically significant.

## Results

### Patient characteristics

A total of 100 Chinese men in Hong Kong with prostate acinar adenocarcinoma were included. The median age of participants was 71 (range 48–81) years old. According to the National Comprehensive Cancer Network guideline on risk stratification and staging criteria [38], these patients were divided into the following groups: localized disease without lymph node metastasis (nine patients with low risk, four patients with intermediate risk, two patients with high risk, 51 patients with very high risk), regional disease with lymph node metastasis (six patients), and distant metastatic disease (28 patients). Other detailed demographic characteristics are summarized in Table 1.

**Table 1:** Clinicopathological characteristics of the Hong Kong and Shanghai cohorts.

Characteristic	Hong Kong cohort (n = 100)	Shanghai cohort (n = 167)
Age of presentation, N (%)		
<50 years	1 (1%)	2 (1.2%)
50–59 years	19 (19%)	36 (21.6%)
60–69 years	27 (27%)	75 (44.9%)
70–79 years	38 (38%)	52 (31.1%)
≥80 years	15 (15%)	2 (1.2%)
Ethnicity		
Han Chinese	100 (100%)	100 (100%)
PSA at diagnosis (ng/mL), N (%)		
<4	3 (3%)	2 (1.2%)
4–9	24 (24%)	16 (9.6%)
10–19	11 (11%)	13 (7.8%)
20–49	14 (14%)	37 (22.2%)
50–99	16 (16%)	22 (13.2%)
≥100	32 (32%)	70 (41.9%)
ISUP Grade Group, N (%)		
1	15 (15%)	4 (2.4%)
2	1 (1%)	7 (4.2%)
3	1 (1%)	14 (8.4%)
4	33 (33%)	40 (24%)
5	50 (50%)	96 (57.5%)
Metastasis status, N (%)		
M0	72 (72%)	61 (36.5%)
M1	28 (28%)	105 (62.9%)
Personal history of cancers	8 (8%)	6 (3.6%)
Family history of cancers*, N (%)		
No	98 (98%)	164 (98.2%)
Unknown	2 (2%)	3 (1.8%)

*Family history of cancers refers to first-degree relatives with prostate cancer only.

### Landscape of germline mutations

Applying filters for quality and rarity in population databases, we identified 60,621 high-confidence coding variants. Next, we conducted in-depth manual analyses of variants in 1,166 genes that included (i) genes associated with hereditary cancers [17,22–24], (ii) cancer-associated genes (oncogenes and tumor suppressor genes based on the Catalogue of Somatic Mutations in Cancer) [25], and (iii) prostate cancer predisposition genes from previously published studies [26–28]. When 60,621 high-confidence coding variants were mapped on the predefined list of 1,166 predisposition genes, 4,205 variants were obtained and annotated according to American College of Medical Genetics and Genomics (ACMG) recommendations [22]. By applying criteria from the ACMG, variants were classified into three categories: either benign and likely benign (benign), variant of uncertain significance (VUS), or pathogenic and likely pathogenic (deleterious). Among 4,205 exonic variants, finally 36 variants (0.9%) were annotated as deleterious, 2,475 variants (58.9%) were VUS, and 1,694 variants (40.3%) were benign (Fig 1).

**Fig 2:**
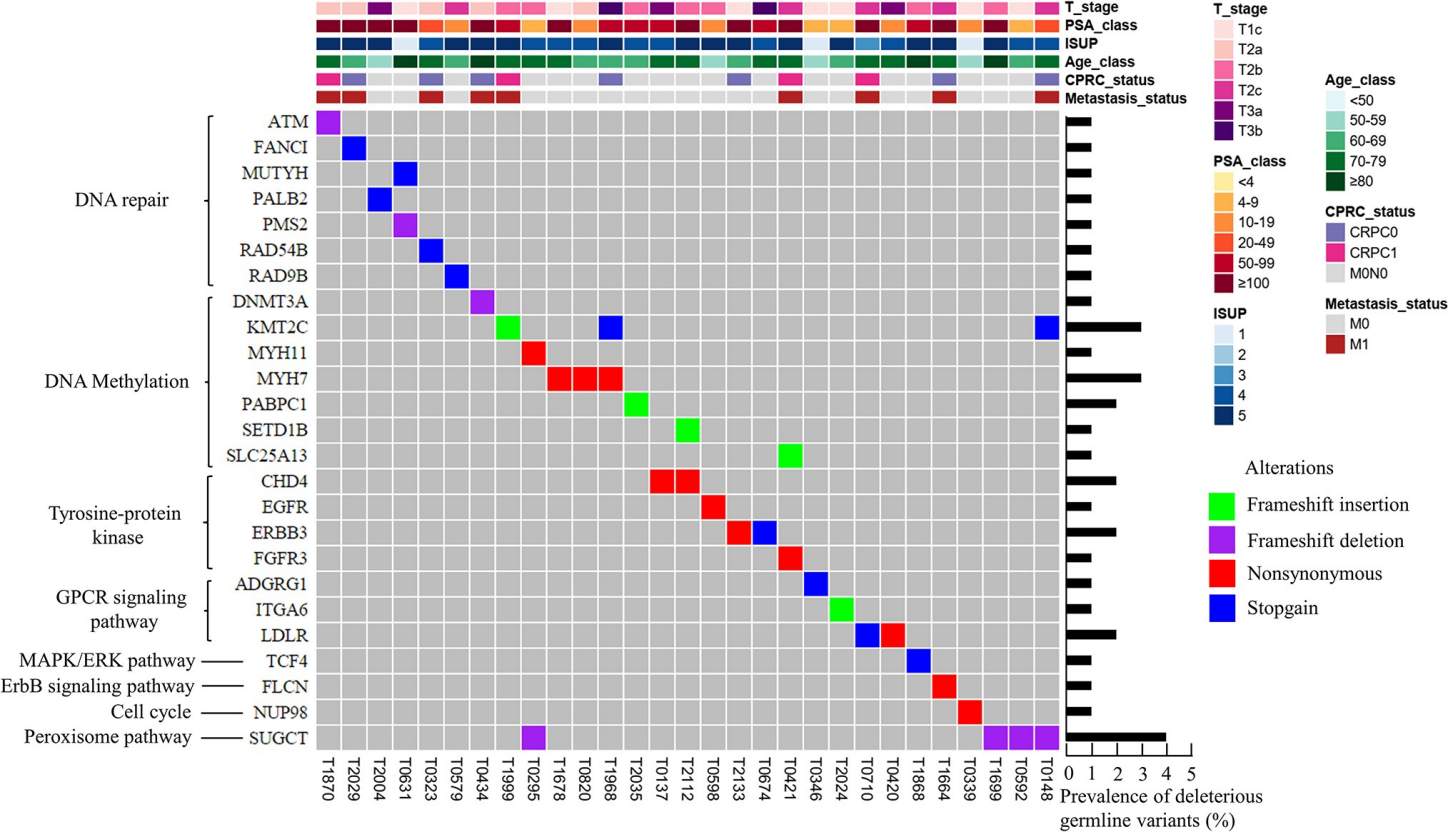
Pathway categorization of deleterious germline variants and clinical characteristics of 29 prostate cancer patients. A total of 36 deleterious variants were identified in 29 patients. Each column corresponds to a patient. The upper section shows the patient’s clinical characteristics [T stage, PSA level, ISUP grade, age, CRPC status and metastasis status]. CRPC1: castration-resistant within one year after starting androgen depletion therapy (ADT); CRPC0: castration-resistant more than one year after starting ADT; M0N0: neither distant metastasis nor regional lymph nodes metastases; M0: no distant metastasis; M1: distant metastasis.

A total of 36 deleterious variants were found in 29 patients (29/100, 29%). A total of seven patients had two deleterious variants. Additionally, two recurrent variants were found in more than one patient: stop-gain variant rs200662726 of lysine methyltransferase 2C (*KMT2C*) and frameshift deletion variant rs752118948 of succinyl-CoA:glutarate-CoA transferase (*SUGCT*), which were identified in two and four patients, respectively (Fig 2). Notably, there was also a frameshift insertion variant detected in *KMT2C*.

**Fig 3:**
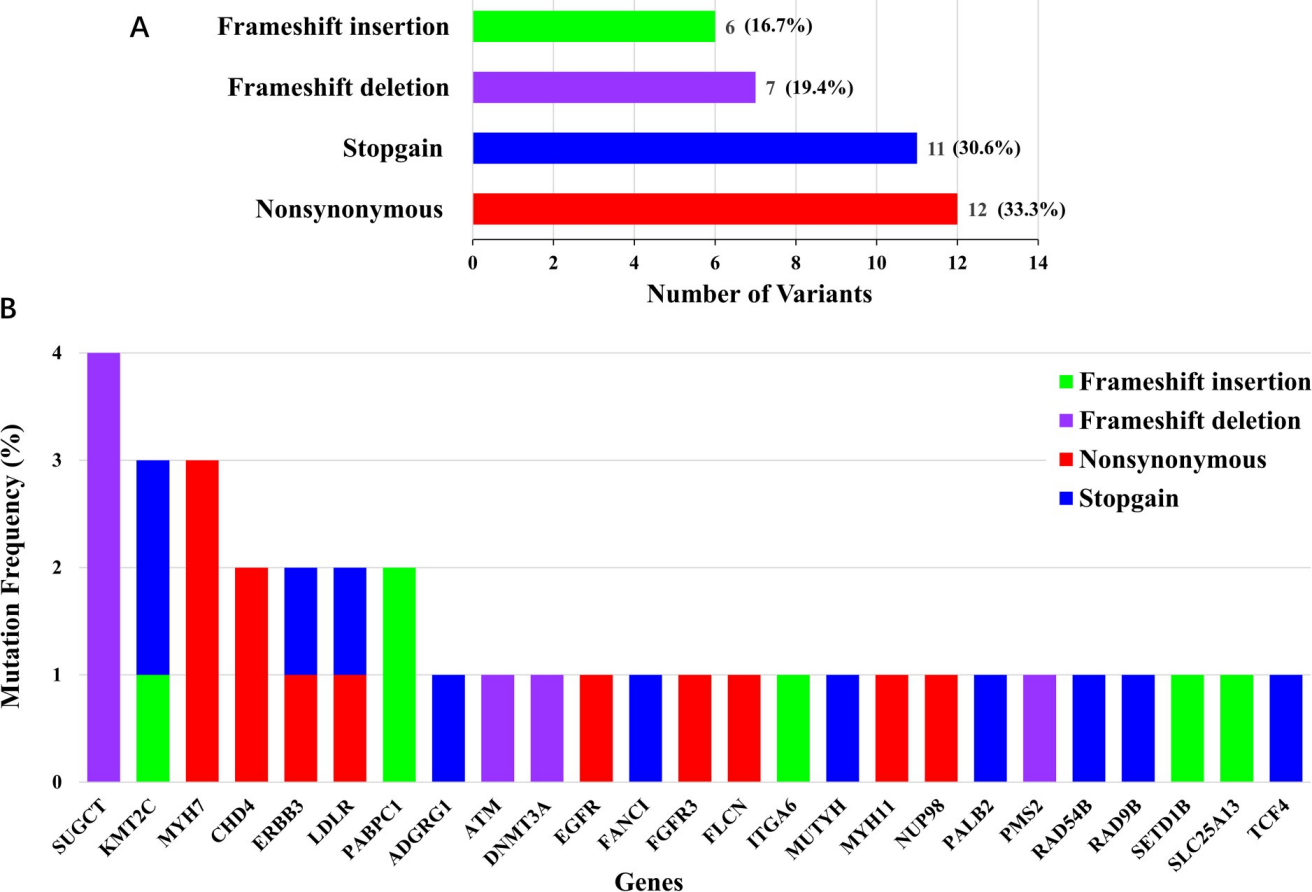
Mutation frequency of deleterious variants and genes. (**A**) The number and frequency of each mutation type among 36 deleterious variants. (**B**) The mutation frequency of 25 deleterious germline genes. Genes are ordered by frequency, and mutations are stratified by mutation type.

Regarding the mutation type of the 36 deleterious variants, 12 (33.3%) variants were nonsynonymous single-nucleotide variant, 11 (30.6%) variants were stop-gain mutations, 7 (19.4%) variants were frameshift deletions, and 6 (16.7%) variants were frameshift insertions (Fig 3A). At the gene level, 36 deleterious variants were identified in 25 genes and *SUGCT* had the largest number of deleterious variants (4%) (Fig 3B).

**Fig 4:**
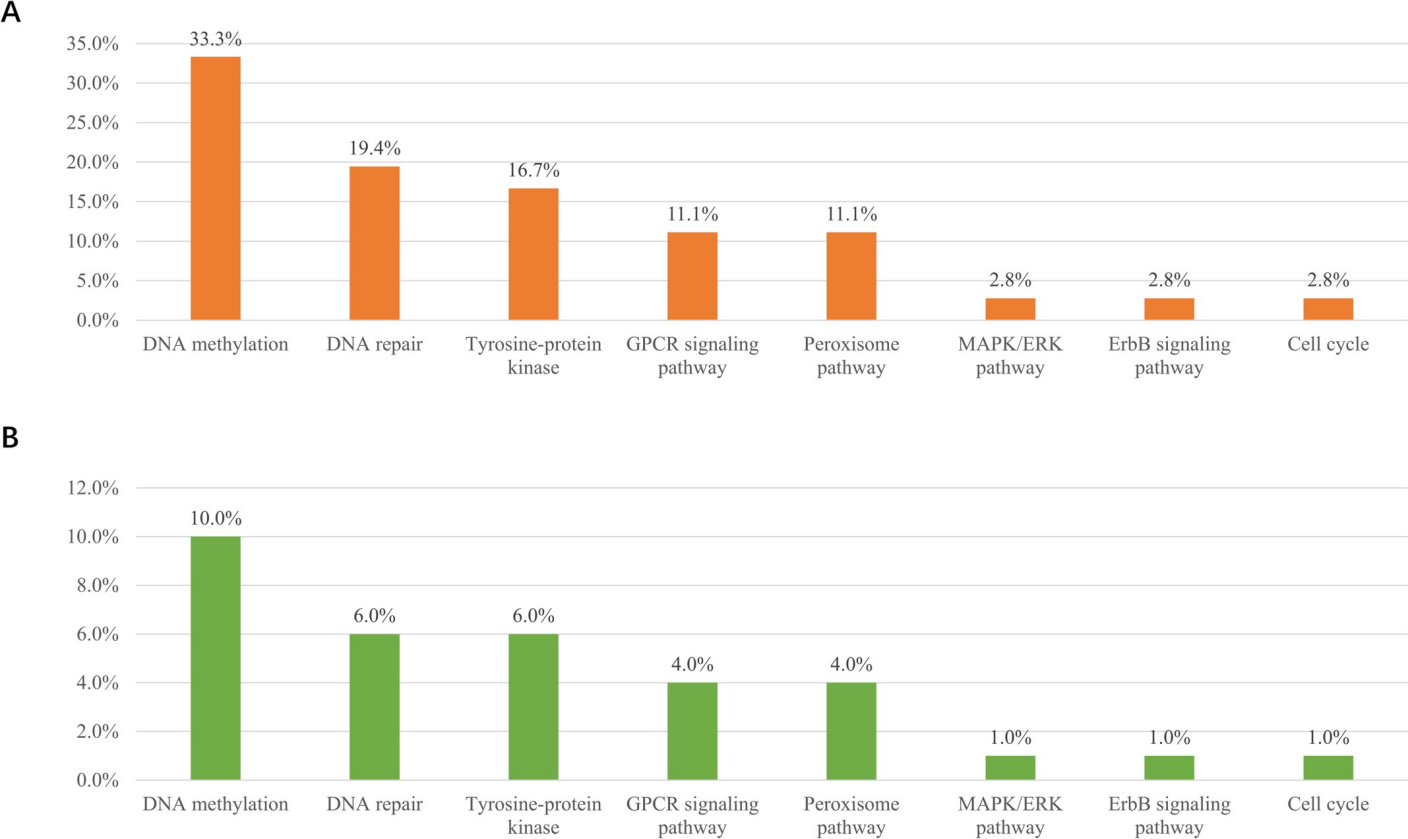
**Categorization of germline mutations among (A) 36 deleterious variants and (B) the patient cohort (N = 100).** The pathways are ordered by frequency.

Concerning the functional categories of the deleterious variants, the largest number of variants were located at genes related to DNA methylation (33.3%, 12/36), followed by variants related to the DDR pathway (19.4%, 7/36) and the tyrosine-protein kinase pathway (16.7%, 6/36). The remaining variants were involved in G-protein-coupled receptor (GPCR) signaling (11.1%, 4/36), peroxisome pathway (11.1%, 4/36), MAPK/ERK pathway (2.8%, 1/36), ErbB signaling pathway (2.8%, 1/36), and cell cycle (2.8%, 1/36) (Fig 4A). Regarding the proportion of the mutations in prostate cancer patients, 10% (10/100) of patients carried variants in DNA methylation pathway, 6% in DDR, 6% in tyrosine-protein kinase pathway, 4% in GPCR signaling and 4% in peroxisome pathway (Fig 4B).

### Comparison of the mutation frequency in prostate cancer patients with that in the general population

**Fig 5:**
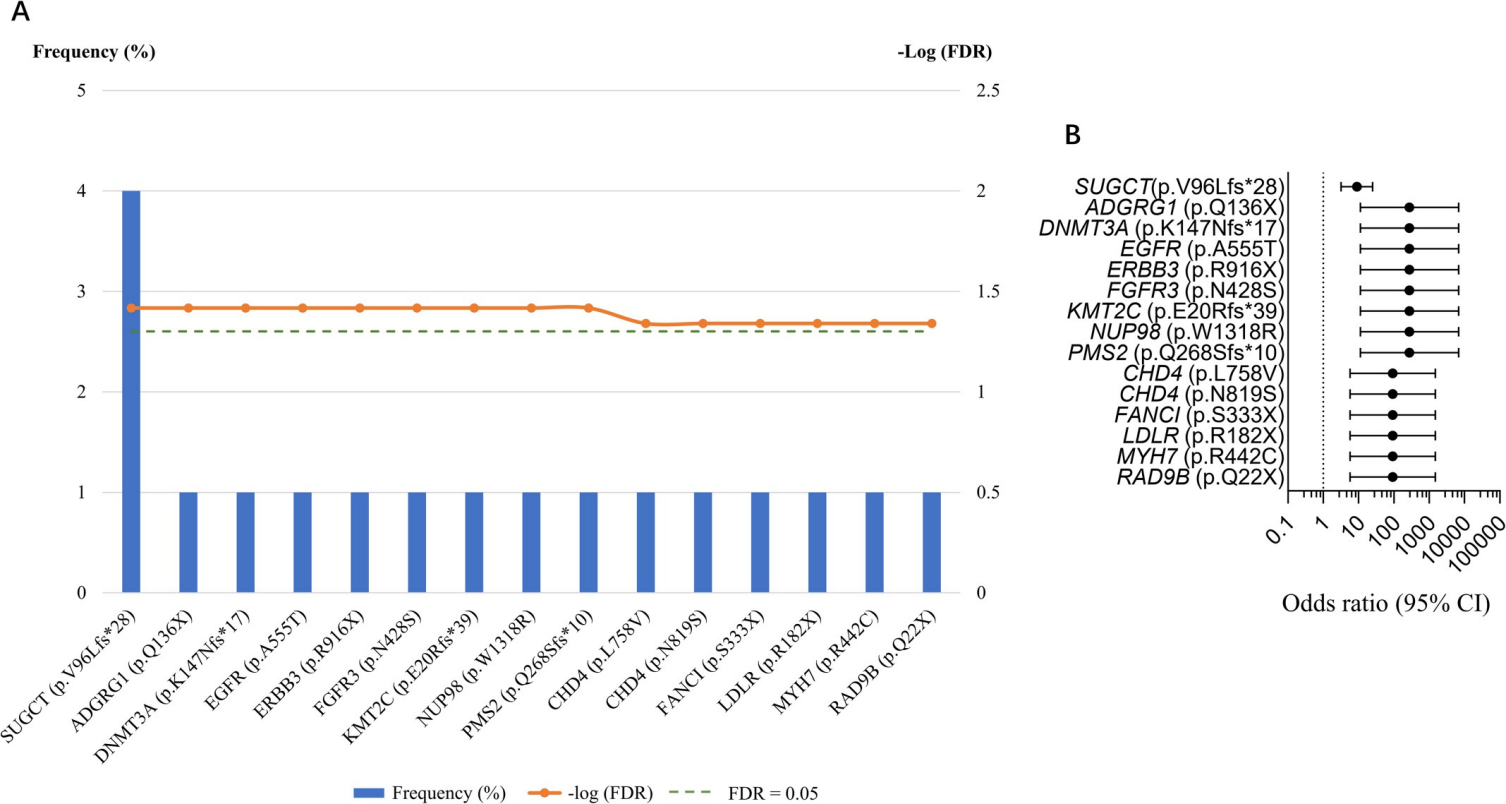
Deleterious germline mutations detected in the Hong Kong cohort compared with East Asian controls. (A) Deleterious germline mutations significantly enriched (False Discovery Rate≤0.05) in the Hong Kong cohort compared with 9,197 East Asian individuals from the GnomAD v2.1.1. (B) Odds ratio of deleterious germline mutations significantly enriched in the Hong Kong cohort compared with 9,197 East Asian individuals from the GnomAD v2.1.1.

To compare the frequencies of deleterious germline variants detected in our cohort with those in the general population, we retrieved data of East Asian individuals from the GnomAD v2.1.1 cohort (n = 9,197 individuals) [19]. By using the cutoff of False Discovery Rate (FDR) ≤ 0.05, 15 out of 36 deleterious variants identified in the Hong Kong cohort were found to have a statistically higher expected frequency compared with GnomAD controls (Fig 5A) (S3 Table). When the odds ratio (OR) and 95% confidence interval (CI) were evaluated for each variant, three identified variants in previously reported DDR genes, *FANCI*, *PMS2* and *RAD9B*, were found to have a higher mutation frequency in our cohort compared with the East Asian GnomAD controls. In addition, all the four tyrosine-protein kinase pathway genes (*CHD4*, *EGFR*, *ERBB3*, and *FGFR3*) and three out of seven DNA methylation pathway genes (*DNMT3A*, *KMT2C*, and *MYH7*) had significantly higher mutation frequency compared with controls. Variants in the GPCR signaling pathway genes *ADGRG1* and *LDLR*, the peroxisome pathway gene *SUGCT*, and the cell cycle pathway gene *NUP98* were also found to be significantly enriched (Fig 5B). To the best of our knowledge, except the three DDR genes, the other 11 genes have not been reported as candidate predisposition genes in Chinese prostate cancer patients (S3 Table). Notably, *CHD4* had two different variants with a much higher frequency, whereas the other genes only had one variant. Four patients in our cohort harbored the p.V96Lfs*28 variant in *SUGCT* and two patients carried the p.R904X variant in *KMT2C*.

### Validation in an independent prostate cancer cohort in Shanghai

By applying the same filters used for the Hong Kong cohort, we validated the previous results using WES data from an independent cohort of 167 Shanghai Chinese prostate cancer patients (S1 Fig). In this cohort, 63% of patients were diagnosed with metastatic prostate cancer upon presentation to the clinic (Table 1). A total of 45 deleterious germline variants located at 28 genes were identified among 42 patients. The percentage of patients with deleterious germline variants in the independent cohort (25.1%) was similar with that in the Hong Kong cohort (29%) (S2A Fig). Additionally, the Hong Kong and Shanghai cohorts had similar mutation types proportion in terms of nonsynonymous (33.3% vs 24.4%) and frameshift deletion (19.4% vs 22.2%) (S2B Fig). Regarding the proportion of deleterious germline variants according to gene function categories, the two cohorts shared three out of the top four categories, which were DNA methylation, DDR, and GPCR signaling pathways (S2C Fig). Seven common deleterious genes were found in both cohorts, namely *FANCI*, *ITGA6*, *LDLR*, *MYH7*, *PABPC1*, *RAD54B* and *SUGCT* (S3A and S3C Fig). Two common deleterious variants rs758404026 (*PABPC1*, p.P446Rfs*30) and rs566695492 (*ITGA6*, p.D114Efs*6) were also present in both cohorts (S3B and S3C Fig).

**Fig 6:**
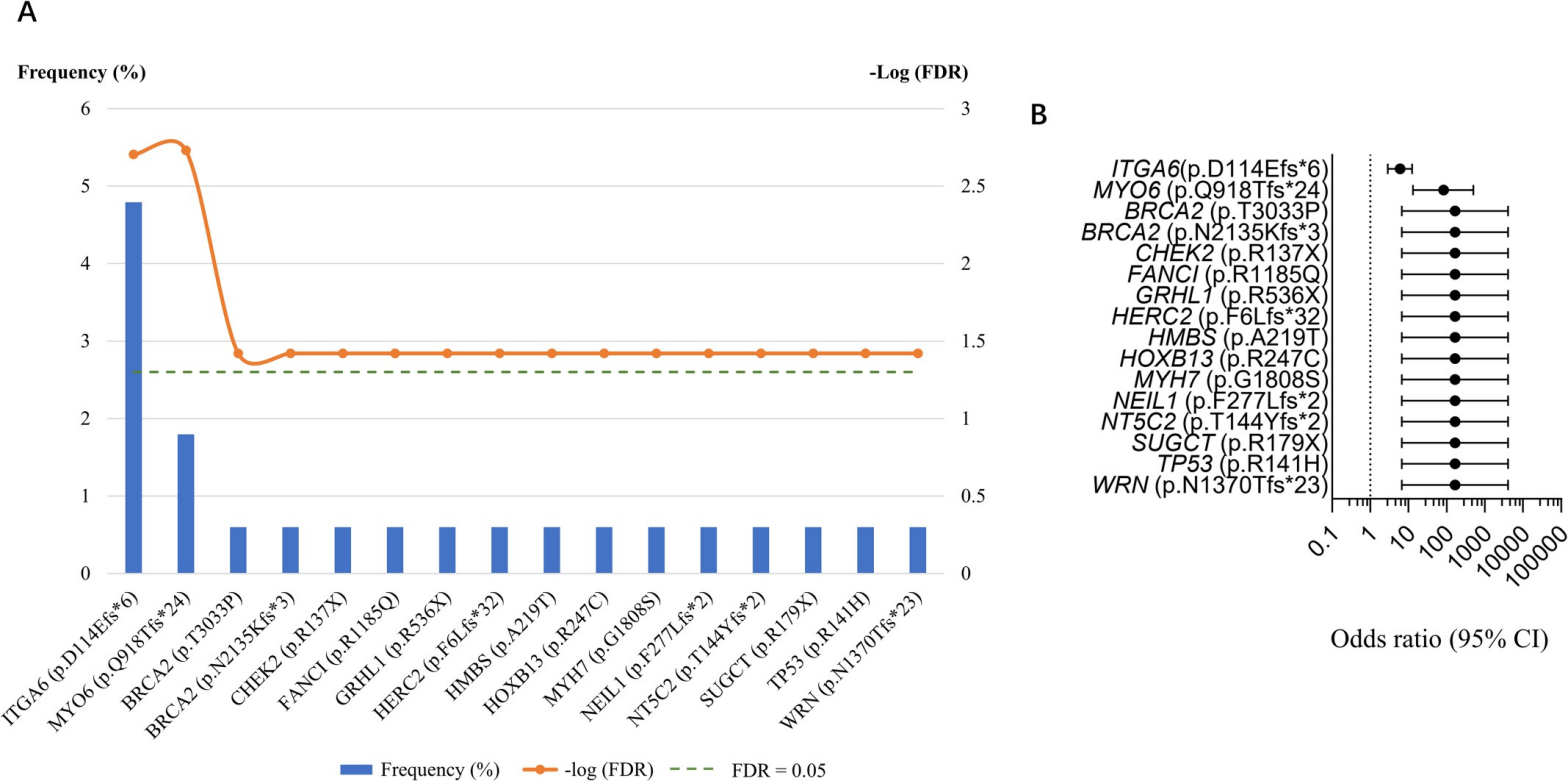
Comparison of the Shanghai cohort and East Asian individuals in the GnomAD. (A) Deleterious germline mutations significantly enriched (False Discovery Rate (FDR)≤0.05) in the Shanghai cohort compared with the 9,197 individuals from the GnomAD v2.1.1. (B) Odds ratio of deleterious germline mutations significantly enriched in the Shanghai cohort compared with the East Asian individuals.

Using the cutoff of FDR ≤ 0.05, 16 deleterious variants in the validation cohort were found to be enriched compared with the GnomAD controls (Fig 6A) (S4 Table). The ORs and 95% CI were calculated for each variant (Fig 6B).

Among all the significant deleterious variants found in the discovery (S3 Table) and validation cohorts (S4 Table), 12 non-DDR novel potential predisposition genes (*ADGRG1*, *CHD4*, *DNMT3A*, *ERBB3*, *GRHL1*, *HMBS*, *LDLR*, *MYH7*, *MYO6*, *NT5C2*, *NUP98* and *SUGCT*) were identified (S3 and S4 Tables), which could be considered as part of the multigene panel for prostate cancer patients, especially Chinese patients.

### Enrichment analysis of VUS-containing genes

During the classification of variants by the ACMG guideline, 684 and 801 VUS-containing genes were detected in the Hong Kong and the Shanghai cohorts, respectively. When these genes were investigated using Gene Ontology (GO) enrichment analysis, the functional term “double-strand break repair” was found to be enriched in both cohorts (S4A and S4B Fig).

Additionally, to further investigate the function of the VUS-containing genes, we performed Kyoto Encyclopedia of Genes and Genomes (KEGG) enrichment analysis. In both cohorts, the analyzed genes were enriched in the PI3K-Akt signaling and prostate cancer pathways (S5A and S5B Fig). In the PI3K-Akt signaling pathway, 46 and 58 VUS-containing genes were enriched in the Hong Kong and Shanghai cohorts, respectively, and among them there were 40 genes in common in both cohorts (S6A Fig, S5 Table). The distribution of VUS-containing genes in the PI3K-Akt signaling pathway (hsa04151) is represented using different colors according to the cohort they were enriched in (S6B Fig). As shown in the figure, many genes associated with cell survival and cell cycle progression were affected. In the prostate cancer pathway, 26 and 32 VUS-containing genes were detected in the Hong Kong and the Shanghai cohorts respectively, with 20 genes shared by both cohorts (S7A Fig, S6 Table). The VUS-containing genes were also highlighted in prostate cancer pathway (hsa05215) (S7B Fig)

To investigate the association between the deleterious-variant-containing genes and the VUS-containing genes in the PI3K-Akt signaling pathway, we constructed a protein-protein interaction network using the STRING database. In the Hong Kong cohort, two categories of genes interacted closely through the nodes of ERBB family (EGFR, ERBB2, and ERBB3) and BRCA1 (S8A Fig). In the Shanghai cohort, two categories of genes were linked closely by the nodes of ERBB family (EGFR, ERBB2 and ERBB3), HRAS, and TP53 (S8B Fig).

Comparing the frequencies of VUS in the PI3K-Akt signaling pathway with that in the general population of East Asian individuals, 57 out of 92 VUS in the Hong Kong cohort (S7 Table) and 77 out of 147 VUS in the Shanghai cohort (S8 Table) were significantly enriched using the cutoff of p-value≤0.05.

Interestingly, among those significant variants (p-value≤0.05), some VUS were located in the same gene, such as the four sites of ERBB2 in the Shanghai cohort and four sites of TSC2 in the Hong Kong cohort. For ERBB2, the 4 variant sites were in two pairs, which were R100Q, R143Q, A466V, and R499Q (S9A Fig, S9 Table). Notably, R100Q and R143Q were both located in linked regions of parallel beta-sheets in receptor L domain (RLD) I (S9B Fig). The other pair was formed by A466V and R499Q (S9B Fig). A466V was located in the N terminal of a beta-strand inside the RLD III, whereas R499Q was located in the linked region of an alpha-helix and a beta-strand. Both RLD I and III were important in the stabilization of ERBB2 homodimer.

In *TSC2*, five significant variants were identified in the Hong Kong cohort. Except R1285Q, the other four variants were in two pairs in the protein structure (S10A and S10D Fig) (S9 Table). All these five variants were located in the dimerization domain. Two pairs of sites contributed to the generation of two small pocket-like motifs (S10B and S10C Fig).

### Association between deleterious-variant-containing genes / VUS-containing genes and clinical outcomes

To investigate the association between the 46 VUS-containing genes in the PI3K-Akt signaling pathway (S5 Table) and the clinical outcomes, using logistic regression we constructed a model to predict the risk of metastasis in the Hong Kong cohort and then validated its performance in the Shanghai cohort.

**Fig 7:**
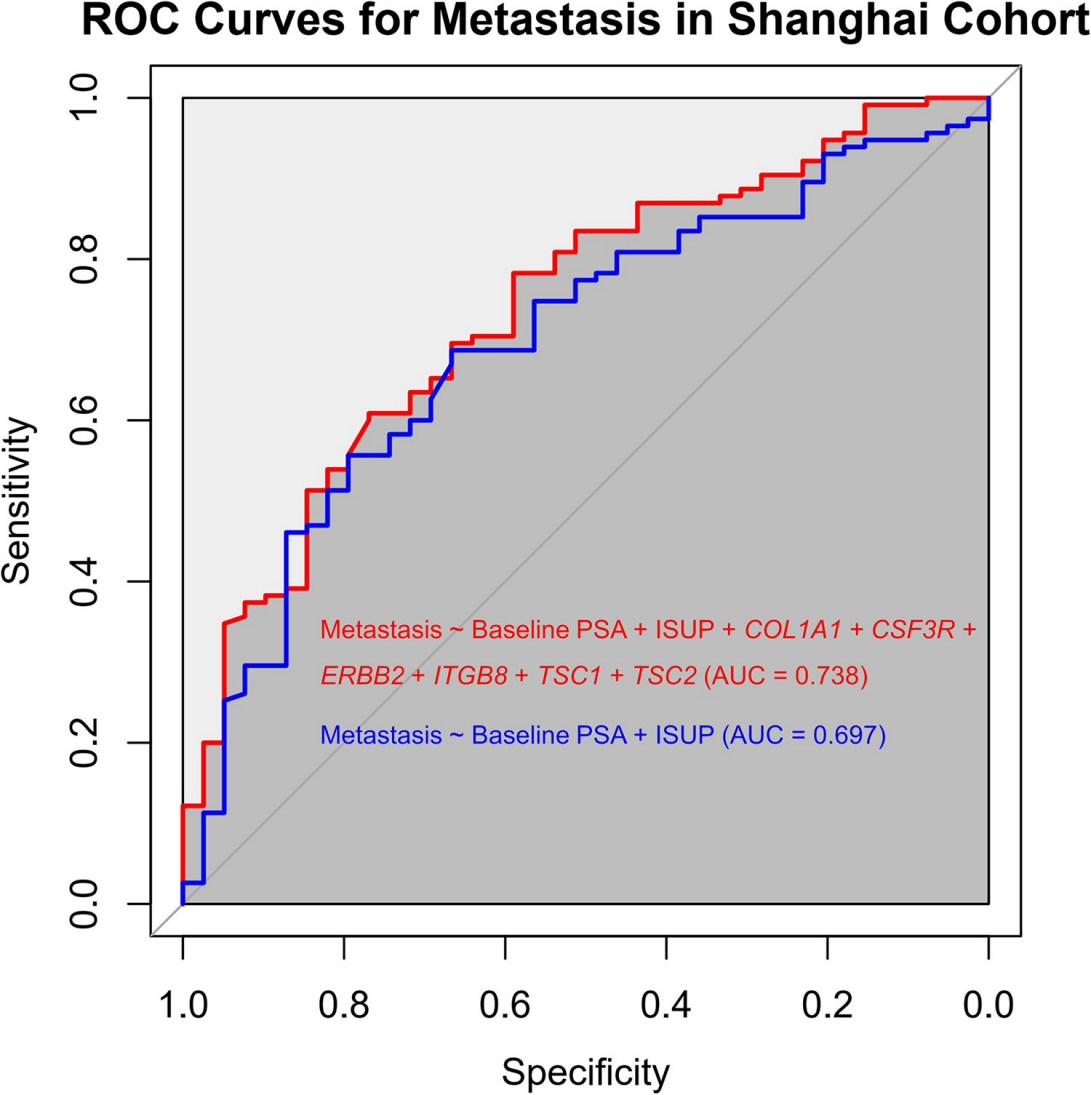
Receiver operating characteristic (ROC) curves for the prediction of metastasis in the Shanghai cohort. The model included baseline Prostate-Specific Antigen (PSA), International Society of Urological Pathology (ISUP) grade, and *COL1A1*, *CSF3R*, *ERBB2*, *ITGB8*, *TSC1* and *TSC2* expression level. The model was built using the Hong Kong cohort by logistic regression, and then validated in the Shanghai cohort. According to the area under the curve (AUC), the constructed model showed a better performance than a control model, including only baseline PSA and ISUP grade.

For metastasis, the best prediction model in the Hong Kong cohort was composed of baseline Prostate-Specific Antigen (PSA), International Society of Urological Pathology (ISUP) grade, *COL1A1*, *CSF3R*, *ERBB2*, *ITGB8*, *TSC1* and *TSC2*. The performance of the model was compared with that of a control model, including only baseline PSA and ISUP grade. The area under the curve (AUC) of the constructed model was 0.927, whereas that of the control model was 0.809, indicating the better performance of the constructed model. This was validated in the Shanghai cohort, in which the constructed model had an AUC of 0.738, whereas that of the control model was 0.697 (baseline PSA and ISUP grade) (Fig 7).

However, we did not find any association between deleterious-variant-containing genes and clinical characteristics including age, ISUP grade, PSA level, metastasis, and castration resistance within one year in the Hong Kong (S11 Fig) and the Shanghai cohort (S12 Fig).

## Discussion

Prostate cancer is one of the most heritable cancer among common cancer types, with approximately 42% of risk attributed to inherited factors [1]. Here, we sequenced germline DNA from 100 Hong Kong Chinese prostate cancer patients using WES and identified many deleterious variants segregated in different genetic pathways. Our findings were further validated in an independent Shanghai Chinese cohort of 167 prostate cancer patients. To the best of our knowledge, our study is the most comprehensive analysis of the germline mutation landscape in Chinese prostate cancer patients to date.

In the Hong Kong cohort, 29% (29/100) of patients harbored 36 deleterious germline mutation and seven patients carried two variants. In the Shanghai cohort, a similar percentage of patients carrying deleterious germline mutation (25.1%, 42/167) was reported, with three patients harboring two variants. In 2020, Wu et al.[16] revealed that 31% of the patients in a 246 Chinese prostate cancer patient cohort harbored a pathogenic germline mutation. The different proportion between our cohort and Wu et al.[16] cohort can be attributed to the different approach used to detect the mutations. Although in this study we sequenced all the germline mutation genes and filtered by a predefined list of 1,166 genes, the cohort of the study by Wu et al.[16] was evaluated using a 276 DDR gene panel for sequencing (S13 Fig and S10 Table). In a cross-sectional study including 3,607 multi-ethnic men with a personal history of prostate cancer and using a 24 genes panel, approximately 17% of patients carried a pathogenic germline variant [14]. The differences between this and our study may be also contributed to the different ethnicity of the population analyzed [39] as well as to the gene panel used (S13 Fig and S10 Table).

Some interesting differences were identified when comparing our results with recent studies regarding the localization of the variants identified. In Nicolosi et al.[14] study, the top 10 genes with pathogenic variants were as follows: *BRCA2* (4.74%), *CHEK2* (2.88%), *ATM* (2.03%), *MUTYH* (2.37%), *APC* (1.28%), *BRCA1* (1.25%), *HOXB13* (1.12%), *MSH2* (0.69%), *TP53* (0.66%), and *PALB2* (0.56%). After comparing the deleterious genes and VUS-containing genes of the Hong Kong and Shanghai cohorts with those in Nicolosi et al.[14] cohort, we found that 6 out of 10 genes were identified in three cohorts. Additionally, three genes harbored a similar mutation proportion, which were *BRCA2* (4.74% vs. 3% vs. 4.19%), *MUTYH* (2.37% vs. 3% vs. 2.4%) and *APC* (1.28% vs. 2% vs. 2.4%) (Nicolosi et al. cohort vs. Hong Kong cohort vs. Shanghai cohort) (S14A Fig and S11 Table).

However, in Nicolosi et al.[14] cohort, *CHEK2* (2.88% vs. 1% vs. 1.2%) harbored a significant higher mutation frequency while *ATM* (2.03% vs. 10% vs. 3.59%), *APC* (1.28% vs. 2% vs. 2.4%) and *MSH2* (0.69% vs. 2% vs. 2.4%) harbored significant lower mutation frequencies compared with the Hong Kong and Shanghai cohorts (Nicolosi et al. cohort vs. Hong Kong cohort vs. Shanghai cohort) (S14A Fig and S11 Table). The majority of races in the Nicolosi et al.[14] cohort (N = 3,607) was white (N = 2,594; 71.9%), followed by Ashkenazi Jewish (N = 234; 6.5%), African American (N = 227; 6.3%), Hispanic (N = 78; 2.2%), Asian (N = 73; 2.0%) and others (N = 401; 11.1%)[14]. Thus, for germline mutations, Chinese population with prostate cancer carried significant different mutation patterns in *CHEK2*, *ATM*, *APC* and *MSH2*, compared to white with prostate cancer.

On the other hand, for the ethnic characteristics in prostate cancer, Chinese population had significant lower incidence and mortality rates compared with white [40]. However, the mortality-to-incidence rate ratio was somewhat higher in China than the Asian average and much higher than the North American average [41]. Moreover, some studies have reported that Asian-Pacific people were more likely to have high-grade prostate cancer than white American,which was not attributed to the later stage of diagnosis [42–44]. Another study indicated that Chinese population was more likely to have poorly differentiated prostate cancer than American and Japanese [45]. These characteristics indicated that Chinese population may have biological differences that increase the susceptibility to have poorly differentiated prostate cancer with lower incidence rate and higher mortality-to-incidence ratio when compared with white.

Therefore, based on the differences in germline mutation frequencies and ethnic characteristics, we speculated that higher mutation frequency of *CHEK2* in white could be the possible biological point for higher incidence rate and higher mutation frequency of *ATM*, *MSH2*, and *APC* in Chinese could be the possible biological reason for more advanced disease and higher mortality-to-incidence ratio.

In the study by Wu et al.[16], *BRCA2* pathogenic variants were the most common identified variants (5.3%), followed by variants in *POLN* (2.4%), *POLG* (1.2%), *ALKBH2* (0.8%), and in other 56 DDR genes. After comparing the deleterious genes and VUS-containing genes of the Hong Kong and Shanghai cohorts with those of the Wu et al.[16] cohort, 11 out of 14 genes were identified in three cohorts and two genes shared a similar mutation frequency, which were *BRCA2* (5.3% vs. 3% vs. 4.19%) and *RAD9B* (0.8% vs. 1% vs. 0.6%) (Wu et al. cohort vs. Hong Kong cohort vs. Shanghai cohort) (S14B Fig and S11 Table).

Seven common deleterious genes were found between the Hong Kong cohort (7/25, 28%) and the Shanghai cohort (7/28, 25%), namely *FANCI*, *ITGA6*, *LDLR*, *MYH7*, *PABPC1*, *RAD54B* and *SUGCT* (S3 Fig). *FANCI* and *RAD54B* were associated with DDR, *MYH7* and *PABPC1* were associated with DNA methylation, and *ITGA6* and *LDLR* were linked to GPCR signaling pathway. Approximately one third of genes were shared between the Hong Kong cohort and Shanghai validation cohort, demonstrating the reliability of the pipeline applied.

In this study, we have also explored the functional enrichment of genes harboring deleterious variants. In 2015, Robinson et al.[12] conducted whole exome and transcriptome sequencing of bone or soft tissue tumor biopsies from a cohort of 150 mCRPC patients. By combining the germline mutation results with the somatic mutation results, they identified the enrichment of six pathways including AR signaling, PI3K, WNT signaling, DNA repair, cell cycle, and chromatin modifier. In the Hong Kong cohort, the deleterious genes were enriched in the following pathways: DNA methylation, DNA repair, tyrosine-protein kinase, GPCR signaling, peroxisome, MAPK/ERK, ErbB signaling, and cell cycle. In the Shanghai validation cohort, deleterious genes were associated with 10 pathways: DNA methylation, DNA repair, tyrosine-protein kinase, GPCR signaling, peroxisome, ILK signaling, hydrolase, chromatin regulation/acetylation, regulation of AR, and regulation of lipid metabolism. Thus, four out of the six enriched pathways reported by Robinson et al.[12] cohort were found in the Hong Kong and the Shanghai cohorts (S2C Fig).

We also identified 12 novel non-DDR predisposition genes (*ADGRG1*, *CHD4*, *DNMT3A*, *ERBB3*, *GRHL1*, *HMBS*, *LDLR*, *MYH7*, *MYO6*, *NT5C2*, *NUP98* and *SUGCT*), which have not been previously reported in prostate cancer patients in all ethnic groups (S3 and S4 Tables). These candidate genes had a higher mutation frequency in the Hong Kong and Shanghai cohorts than the East Asian controls with an OR≥1 and FDR≤0.05. Two novel predisposition gene *MYH7* and *LDLR* belong to the ACMG published recommendations for reporting secondary findings in clinical exome and genome sequencing (ACMG SF v2.0)[22] (N = 59) (S1 Table) while *DNMT3A*, *CHD4*, *ERBB3*, *NT5C2*, and *NUP98* belong to the panel of 716 cancer driver genes from TCGA and ICGC identified by the platform OncoVar [46] (S2 Table). Furthermore, they were enriched in eight different pathways (DNA methylation, tyrosine-protein kinase, GPCR signaling, regulation of lipid metabolism, ILK signaling, hydrolase, peroxisome, and cell cycle), which did not include the DDR pathway.

Interestingly, 3 out of 12 novel non-DDR genes (*DNMT3A*, *HMBS* and *MYH7*) were related to DNA methylation that contributes significantly to the development and progression of prostate cancer [47–50]. Not only significant changes of DNA methylation are observed between normal prostate and prostate cancer tissue [51,52], but also the changes of DNA methylation are associated with carcinogenesis and progression of prostate cancer by silencing tumor-suppressor genes, activating oncogenic drivers, and driving therapy resistance [53]. Moreover, interplay among DNA methylation, cancer metabolism and androgen receptor regulation has been reported to play an important role in prostate cancer [53]. Recently, a study revealed a novel epigenomic subtype associated with hypermethylation and somatic mutations in *TET2*, *DNMT3B*, *IDH1* and *BRAF* by whole-genome bisulfite sequencing paired with whole-genome and transcriptome sequencing of 100 castration-resistant prostate metastases [54]. Of note, *DNMT3A* found in our study is the close and important paralog of *DNMT3B*, both of which are DNA methyltransferases playing an essential role in DNA methylation [55,56].

For the other two novel non-DDR genes, *CHD4* and *ERBB3* were involved in the tyrosine-protein kinase pathway. Although it is rare to observe dominant mutations of tyrosine kinases in the oncogenic alterations of prostate cancer [57], we should not overlook the importance of tyrosine-protein kinase pathway. Members of nonreceptor tyrosine kinase (NRTK) including *Src*, *FAK*, *JaK1/2*, and *ETK* were involved in the cell proliferation, migration, invasion, angiogenesis, and apoptosis of prostate cancer [58]. Moreover, *Src* has been reported to promote CRPC through the regulation of canonical and non-canonical AR binding site associated genes [59]. Of note, *Src* could enhance *ERBB2*/*ERBB3* signaling and biological functions through positively modulating *ERBB2* and *ERBB3* heterocomplex formation and function [60]. Recently, a study revealed that somatic mutation frequencies of *FOXA1*, *ZNF292* and *CHD1* in Chinese patients were remarkedly higher than those of Western cohorts by whole-genome, whole-transcriptome and DNA methylation data of 208 pairs of tumor tissues and matched healthy control tissues from patients with primary prostate cancer [51]. Therefore, the importance of somatic mutation of *CHD1* and germline mutation of *CHD4* has been demonstrated in Chinese patients with prostate cancer compared to Western cohorts, highlighting the ethnic characteristics of CHD family genes in Chinese population.

Another two novel non-DDR genes, *ADGRG1* and *LDLR* were related to GPCR signaling pathway, which is known to play a vital role in cancer initiation and progression, including tumor growth, invasion, migration and metastasis [61]. A variety of GPCRs related with reproductive function have been reported to be implicated in the oncogenesis and progression of prostate cancer, including gonadotropin-releasing hormone (GnRH) receptor, luteinizing hormone receptor, follicle-stimulating hormone receptor, relaxin receptor, ghrelin receptor, and kisspeptin receptor [62]. By specifically blocking the GnRH receptor, a GPCRs targeted drug, Degarelix, a FDA approved drug, is applied to treat advanced prostate cancer by decreasing the amount of testosterone [63]. Another GPCR signaling pathway related gene, *LDLR*, whose mutation type has been demonstrated to be closely related with the phenotype of familial hypercholesterolemia [64], was reported to be associated with higher Gleason grade in prostate cancer [65].

To date, the multigene panel for Chinese prostate cancer patients is based on the genetic knowledge from the European and American populations. However, the differences in genetic background among ethnic groups may affect its efficacy in clinical practice. Moreover, the current multigene panel for prostate cancer contains primarily DDR genes involved in homologous recombination and mismatch repair. Nonetheless, susceptibility genes from other functional pathways could also be involved in the pathogenesis and development of prostate cancer. Therefore, these 12 novel non-DDR predisposition genes (S3 and S4 Tables) could be considered as part of the multigene panel for prostate cancer patients, especially Chinese patients.

Apart from the analysis of deleterious variants, we studied VUS, which were thought to have uncertain significance in the annotation step according to the ACMG guidelines. Recently, Federici and Soddu reviewed the studies in hereditary breast and ovary cancers and highlighted the need to seek easily applicable ways to accurately classify VUS, as well as to increase the amount of usable information from next generation sequencing data [66]. In our study, using GO and KEGG enrichment analysis of VUS-containing genes in both cohorts, we consistently found DDR related terms, PI3K-Akt signaling pathway, and prostate cancer pathway.

Several studies have demonstrated that somatic mutations in the PI3K-Akt pathway could coordinate PTEN [67], mTOR [68], AR, MAPK, Wnt [69] and TGF-β signaling pathways [70] to play an important role in the tumorigenesis, progression, and treatment in prostate cancer. However, except one study by Robinson et al.[12], there are no available studies on the landscape of germline mutations in genes of PI3K-Akt pathway. Robinson et al.[12] reported that mCRPC harbored genomic alterations of driver genes in the PI3K pathway, such as *AKT1*, *PTEN*, *PIK3CA*, *PIK3CB*, and *PIK3R1*. Additionally, another study reported that mutations in *PIK3CA* were correlated with poor survival in prostate cancer [71]. Of note, in the study by Robinson et al.[12], PI3K-Akt pathway was listed as one of the top enriched pathways according to KEGG enrichment analysis of a list of 13,972 mutated genes (S12 Table) (S15A Fig). Here, 281 genes were found in the PI3K-Akt pathway, which were overlapping with most of VUS-containing genes of the PI3K-Akt pathway found in the Hong Kong (N = 46) and Shanghai cohorts (N = 58) (S15B Fig and S13 Table). Given that the study by Robinson et al.[12] investigated the genomic alterations in Caucasian population, our study was the first one to reveal the germline mutation landscape in PI3K-Akt signaling pathway in Chinese prostate cancer patients.

Protein-protein interaction networks analysis of deleterious genes and the VUS-containing genes in the PI3K-Akt signaling pathway revealed that these two groups of genes interacted closely with each other in both cohorts by the nodes of ERBB family (EGFR, ERBB2 and ERBB3), BRCA1, HRAS, and TP53. A recent study revealed that the combined protein expression patterns of EGFR, ERBB2, and ERBB3 were associated with a higher risk of progression and mortality in prostate cancer [72]. HRAS, as one of the Ras oncogene family, was found to have increased amplification rate in hormone-resistant prostate cancer compared with hormone-sensitive prostate cancer [73]. Finally, several studies have shown that TP53 is one of the most commonly mutated genes in primary prostate cancer and that it plays a crucial role in the development and progression of prostate cancer [74,75].

When comparing the frequencies of VUS in the PI3K-Akt signaling pathway with that in the East Asian population, 57 VUS in the Hong Kong cohort (S7 Table) and 77 VUS in the Shanghai cohort (S8 Table) were found to significantly differ (P≤0.05). Investigating the impact of such variants on the protein structure, we found that in ERBB2, one pair of variants located at RLD I and the other pair was near RLD III, which are important domains for the stabilization of ERBB2 homodimer (S9 Table)[76]. As for TSC2, the four significant variants were distributed and located to the dimerization domain, contributing to generate two small pocket-like motifs (S9 Table). Of note, alanine (A) to valine (V) and proline (P) to leucine (L) mutations were not associated with a change in amino acid property [77,78], whereas serine (S) to valine (V) mutation resulted in a change from a polar uncharged side chain to an hydrophobic chain, which would possibly affect the structure of the small pocket-like motif of A1235V and S1222V. None of these variants was recorded as natural variant in UniProt database [79]. Besides, there was a significant deletion of S1440_D1446 located at the proximity of the C-terminal of the dimerization domain (S9 Table). Therefore, we considered that all the variants and the deletion could result in possible dysfunction of the dimerization domain of TSC2, which may be related to carcinogenesis [80,81].

Regarding the clinical factors and the deleterious-variant-containing genes, we did not observe a relationship between the presence of deleterious-variant-containing genes and clinical characteristics. However, by using logistic regression strategy, we found and validated that several VUS-containing genes (*COL1A1*, *CSF3R*, *ERBB2*, *ITGB8*, *TSC1* and *TSC2*) in the PI3K-Akt signaling pathway can improve the predicting of metastasis in prostate cancer patients in Hong Kong and Shanghai cohorts.

There were several strengths of this study. First, the sample size of this study was comparable to prior studies. We included 100 patients in the primary cohort and 167 patients in the validation cohort. Second, we had an independent cohort from Shanghai for validation, making the results more reliable. Finally, the most updated pipeline and variant annotation information form the ACMG were applied in this study.

Although we identified that some variants are pathogenic for the protein they code for and absent in a set of control individuals, more investigations are needed to confirm their substantial implications in prostate cancer.

In conclusion, we dissected the comprehensive germline mutation landscape of Chinese prostate cancer patients using WES and identified 36 deleterious variants in 25 genes that are enriched in eight functional pathways. These findings were validated in an independent cohort of 167 patients. Of note, 12 novel predisposition genes were identified that have not been previously reported in prostate cancer patients in all ethnic. Moreover, by investigating the information of VUS, a group of mutation genes in PI3K-Akt pathway were consistently detected in both cohorts. Besides, a logistic regression model for metastasis prediction using the expression levels of such genes was built. Together, these findings help to unveil the comprehensive germline mutation profile of Chinese prostate cancer and could be helpful for the optimization of cancer screening as well as risk assessment and development of personalized therapy.

## Supporting information

S1 Fig Overview of the data analysis strategy to identify candidate prostate cancer susceptibility genes in the validation cohort. A total of 167 patients with prostate cancer were included. Germline samples were whole exome–sequenced and aligned to human genome assembly hg19 before variant calling and annotations. All germline variants were identified and filtered by (i) MAF < 0.01 in any East Asian population database including ExAC, 1000 Genomes and GnomAD v2.1., (ii) variants in the exonic region, (iii) variants with total coverage > 20x, (iv) variants with Fisher score > 60, and (v) VAF ≥ 25%. Among the 105,504 variants after filtering, 7,510 variants belonging to a predefined list of 1,166 genes were annotated as pathogenic or likely pathogenic (deleterious), variant of uncertain significance (VUS), likely benign, or benign (benign) according the ACMG guidelines. The 1,166-gene list is provided in S1 Table. MAF, minor allele frequency; VAF, variant allele frequency/fraction.
(TIF)

S2 Fig Comparison of the deleterious germline mutation frequency in the Hong Kong and Shanghai cohorts. **(A)** Overall proportion of deleterious germline variants among prostate cancer patients. Overall proportion of deleterious germline variants according to **(B)** mutation types and **(C)** gene function categories.
(TIF)

S3 Fig Comparison of common deleterious genes/variants between the Hong Kong and Shanghai cohorts. Number of common deleterious **(A)** genes and **(B)** variants in both cohorts. **(C)** Details of common deleterious genes/variants. Common variants are shown in red. The (#) following the variant name represents the number of the variant detected in the cohort. N.A. indicates “not available.”
(TIF)

S4 Fig **Gene Ontology enrichment analysis of variants of uncertain significance (VUS)-containing genes in the (A) Hong Kong and (B) Shanghai cohorts.** The functional categories of GO enrichment were subsequently ranked by the gene ratio (x-axis), which was the percentage of the number of genes present in this GO term over the total number of genes in this category.
(TIF)

S5 Fig **Kyoto Encyclopedia of Genes and Genomes (KEGG) enrichment analysis of variants of uncertain significance (VUS)-containing genes in (A) the Hong Kong and (B) Shanghai cohorts.** Top 20 KEGG pathways were ranked by gene ratio (x-axis), which was the percentage of identified genes over the total genes of a given pathway/term.
(TIF)

S6 Fig PI3K-Akt signaling pathway (hsa04151) highlighted with variants of uncertain significance (VUS)-containing genes from the Hong Kong and Shanghai cohorts. (**A**) The overlapping VUS-containing genes in PI3K-Akt signaling pathway in two cohorts were summarized in the Venn diagram. (**B**) VUS-containing genes appeared in both cohorts, in only Hong Kong cohort and in only Shanghai cohort were highlighted in green, gray, and red, respectively.
(TIF)

S7 Fig Prostate cancer pathway (hsa05215) highlighted with variants of uncertain significance (VUS)-containing genes from the Hong Kong and Shanghai cohorts. (**A**) The overlapping VUS-containing genes in prostate cancer pathway in the two cohorts are summarized in the Venn diagram. (**B**) VUS-containing genes that appeared in both cohorts, in only Hong Kong cohort and in only Shanghai cohort are highlighted in green, gray, and red, respectively.
(TIF)

S8 Fig **Protein-protein interaction network between proteins encoded by the deleterious-variant-containing genes and the variants of uncertain significance (VUS)-containing genes in the PI3K-Akt signaling pathway in the (A) Hong Kong and (B) Shanghai cohorts.** Proteins encoded by both categories, by only deleterious-variant-containing genes, and by only VUS-containing genes in the PI3K-Akt signaling pathway are highlighted in red, blue, and green, respectively. The size of rectangle and font size of protein name are represented depending on the number of edges (connectivity/degree) that each node (protein) has. The more edges, the bigger the node and the font size, the more connective the protein is. The active interaction source is experiments only. The thickness of the edges is represented the strength of data support. The thicker the edges, the more strength the experiments support.
(TIF)

S9 Fig Structure of the extracellular domain encompassing amino acids 23–629 of receptor tyrosine-protein kinase ERBB2 and location of the four variants. The structure model of ERBB2 (GenBank accession: NM_001289937) was generated using the online tool SWISS-MODEL (https://swissmodel.expasy.org/) with the template of Cryo-EM structure of Receptor tyrosine-protein kinase erbB-2 (SMTL ID: 6bgt.1.A). (**A**) Four variants in two pairs (closely located within 50 amino acids) were marked and linked, namely R100Q and R143Q (with a molecular distance of 8.31 Å); A466V and R499Q (16.97 Å). (**B**) The locations of the four variants among the domains of ERBB2.
(TIF)

S10 Fig Structure of TSC complex subunit 2 (TSC2) and location of the four variants. The structure model of TSC2 (GenBank accession: NM_001318831) was generated using the online tool SWISS-MODEL (https://swissmodel.expasy.org/) with the template of Cryo-EM structure of human TSC complex (SMTL ID: 7dl2.1). (**A**) Four variants in two pairs (closely located within 50 amino acids) were marked and linked, from left to right, A1235V and S1222L, P1071L and A1079V. (**B**) Structure model of the pair sites A1235V and S1222L (separated by 12.67 Å). (**C**) Structure model of the pair sites P1071L and A1079V (6.71 Å). (**D**) Locations of the four variants among the domains of TSC2.
(TIF)

S11 Fig Association between deleterious-variant-containing genes and clinical outcomes in the Hong Kong cohort. **(A)** Risk of deleterious-variant-containing genes based on clinical characteristics (metastasis and castration resistance within one year) were calculated. **(B, C, and D)** The difference of clinical characteristics (age, ISUP grade, and PSA) between the deleterious-variant-containing genes carriers and the non-carriers were analyzed. “ns” indicates “not significant.”
(TIF)

S12 Fig Association between deleterious-variant-containing genes and clinical outcomes in the Shanghai cohort. **(A)** Risk of deleterious-variant-containing based on clinical characteristics (metastasis and castration resistance within one year) were calculated. **(B, C, and D)** The difference of clinical characteristics (age, ISUP grade, and PSA) between the deleterious-variant-containing genes carriers and the non-carriers were analyzed. “ns” indicates “not significant.”
(TIF)

S13 Fig Venn diagram of the gene panels from our study and other two studies. The gene panels from our study, those from the study by Wu et al.[16], and those from the study by Nicolosi et al.[14] are shown in yellow, blue, and green, respectively. The gene names of the gene panels are listed in S10 Table.
(TIF)

S14 Fig Summary of the gene mutation frequency in the three cohorts compared. In the Hong Kong and Shanghai cohorts, the mutated genes included those with a deleterious variant and the variant of uncertain significance (VUS)-containing genes. **(A)** Comparison of the frequency of the top 10 genes from Nicolosi et al.[14] cohort in the Nicolosi et al. cohort, the Hong Kong cohort, the Shanghai cohort, as well as the combined Hong Kong and Shanghai cohort. **(B)** Comparison of the frequency of the top 10 genes from Wu et al.[16] cohort in the Wu et al. cohort, the Hong Kong cohort, the Shanghai cohort, as well as the combined Hong Kong and Shanghai cohort.
(TIF)

S15 Fig KEGG enrichment analysis of the 13,972 variant-containing genes from the study of Robinson et al.[12]. (A) Top 20 KEGG pathways were showed after analyzing 13,972 variant-containing genes (S12 Table) from the study of Robinson et al.[12] (B) Venn diagram of the genes in the PI3K-Akt pathway from the study by Robinson et al.[12] (red), the Hong Kong cohort (green) and the Shanghai cohort (blue). The frequency of the genes in the PI3K-Akt pathway from three cohorts is listed in S13 Table.
(TIF)

S1 Table Summary of predefined predisposition gene panel of 1,166 genes. (XLSX)

S2 Table Panel of 716 cancer driver genes from The Cancer Genome Atlas (TCGA) and International Cancer Genome Consortium (ICGC) identified by the platform OncoVar. (XLSX)

S3 Table Details of 36 deleterious germline variants identified in the Hong Kong cohort. (XLSX)

S4 Table Details of 45 deleterious germline variants identified in the Shanghai cohort. (XLSX)

S5 Table Summary of the VUS-containing genes in the PI3K-Akt signaling pathway enriched in the Hong Kong (N = 46) and Shanghai (N = 58) cohorts. (XLSX)

S6 Table Summary of the VUS-containing genes in the prostate cancer pathway enriched in the Hong Kong (N = 26) and Shanghai (N = 32) cohorts. (XLSX)

S7 Table Variants of PI3K-Akt pathway in the Hong Kong cohort (sorted by p value). (XLSX)

S8 Table Variants of PI3K-Akt pathway in the Shanghai cohort (sorted by p value). (XLSX)

S9 Table Summary of variants in TSC2 and ERBB2. (XLSX)

S10 Table Summary of gene panels in three studies. (XLSX)

S11 Table Summary of frequency of variants detected in cohorts. (XLSX)

S12 Table Gene list of 13,972 variant-containing genes from the study of Robinson et al. [12]. (XLSX)

S13 Table Frequency of the VUS-containing genes in the PI3K-Akt signaling pathway enriched in the Hong Kong cohort, the Shanghai cohort and variant-containing genes in the PI3K-Akt signaling pathway enriched in the study of Robinson et al. [12]. (XLSX)

S14 Table Sequencing Coverage and Quality Statistics of Whole exome sequencing (WES). (XLSX)
