# Supplementary figures and images for: Whole-exome sequencing reveals a comprehensive germline mutation landscape and identifies twelve novel predisposition genes in Chinese prostate cancer patients
Source: PLoS Genet. 2022 Sep 12;18(9):e1010373. doi: 10.1371/journal.pgen.1010373 (PMC9499300; doi:10.1371/journal.pgen.1010373)

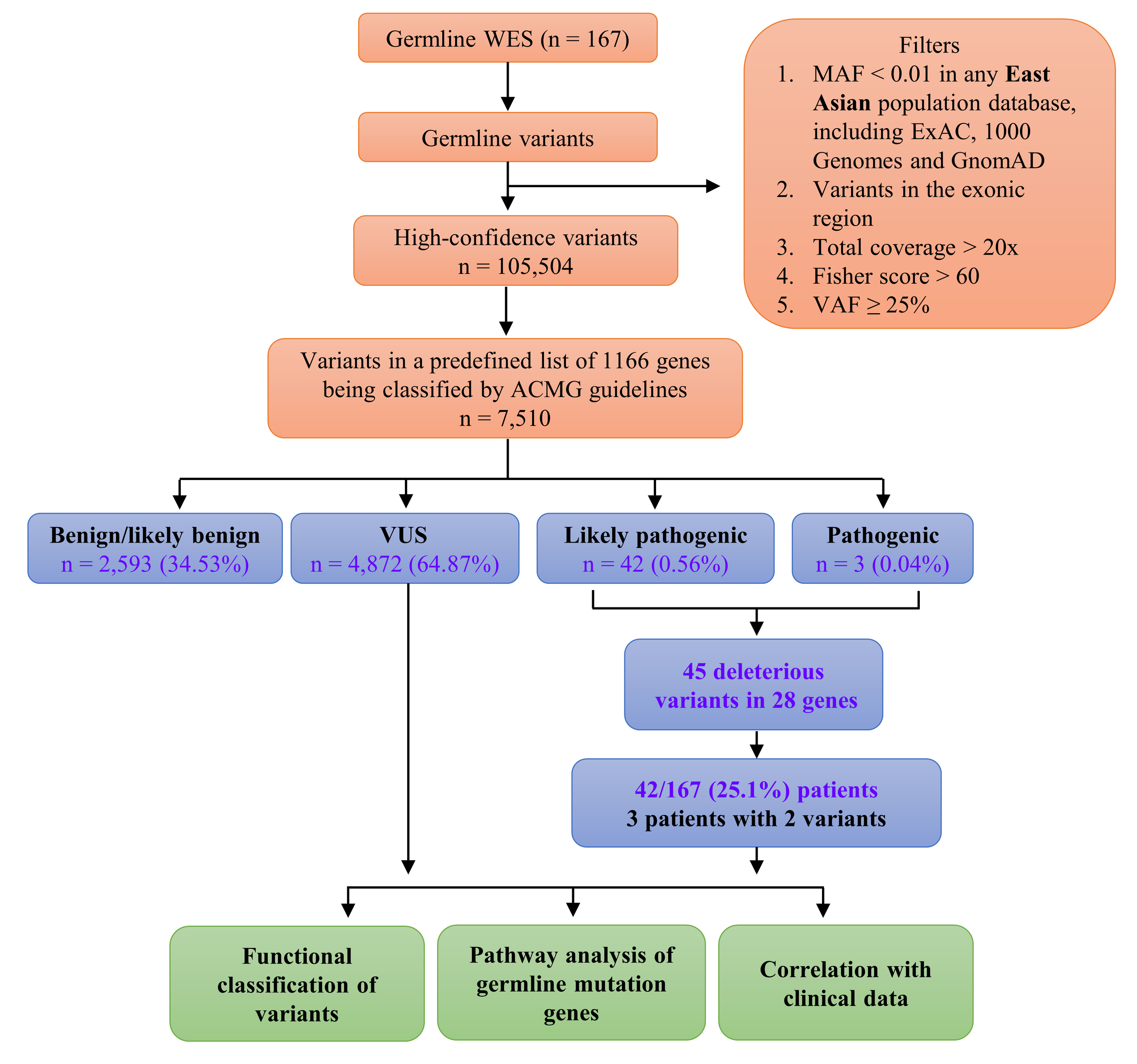

Supplement: S1 Fig — A total of 167 patients with prostate cancer were included. Germline samples were whole exome–sequenced and aligned to human genome assembly hg19 before variant calling and annotations. All germline variants were identified and filtered by (i) MAF < 0.01 in any East Asian population database including ExAC, 1000 Genomes and GnomAD v2.1., (ii) variants in the exonic region, (iii) variants with total coverage > 20x, (iv) variants with Fisher score > 60, and (v) VAF ≥ 25%. Among the 105,504 variants after filtering, 7,510 variants belonging to a predefined list of 1,166 genes were annotated as pathogenic or likely pathogenic (deleterious), variant of uncertain significance (VUS), likely benign, or benign (benign) according the ACMG guidelines. The 1,166-gene list is provided in S1 Table. MAF, minor allele frequency; VAF, variant allele frequency/fraction. (TIF) [file pgen.1010373.s001.tif]

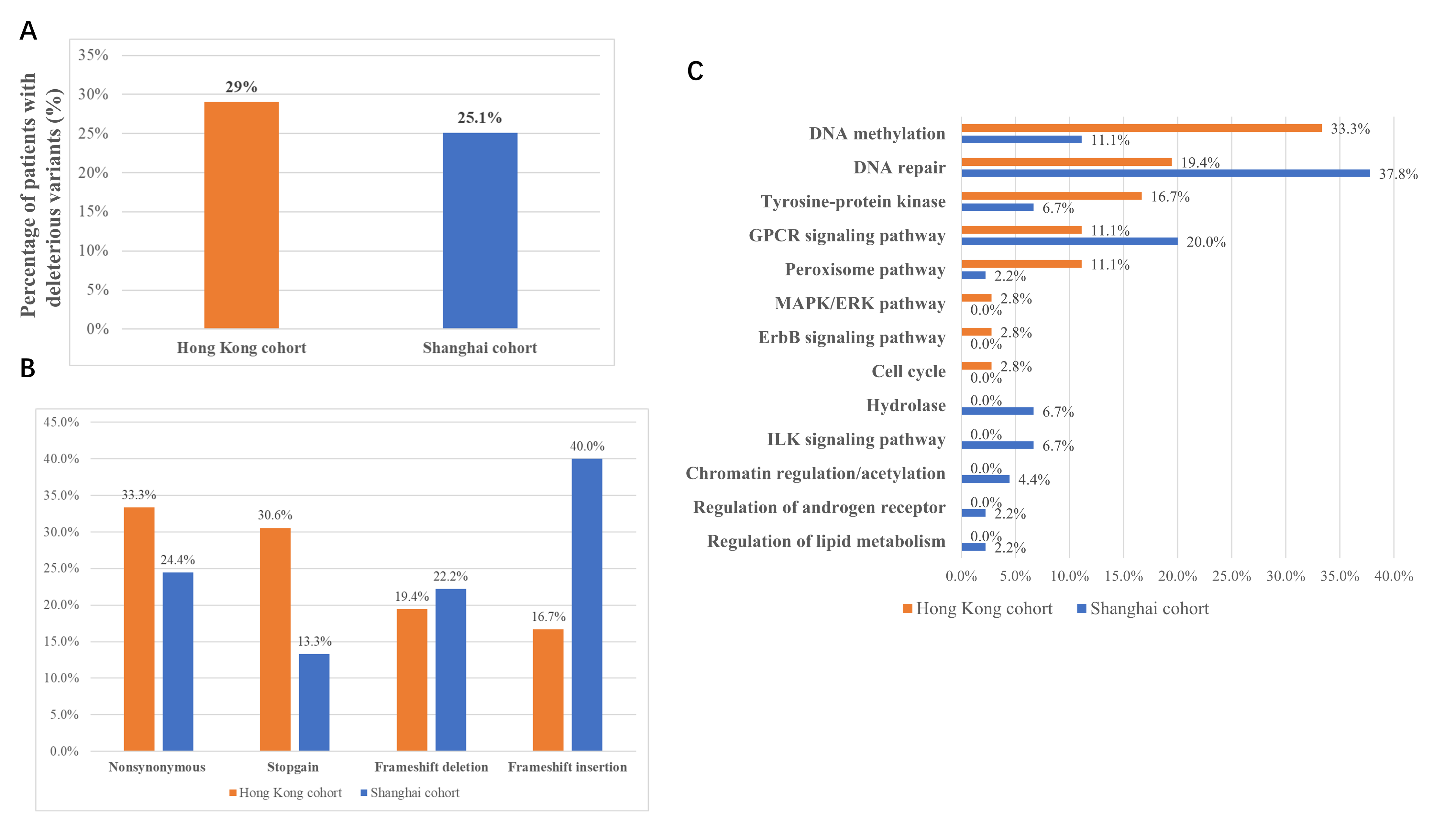

Supplement: S2 Fig — (A) Overall proportion of deleterious germline variants among prostate cancer patients. Overall proportion of deleterious germline variants according to (B) mutation types and (C) gene function categories. (TIF) [file pgen.1010373.s002.tif]

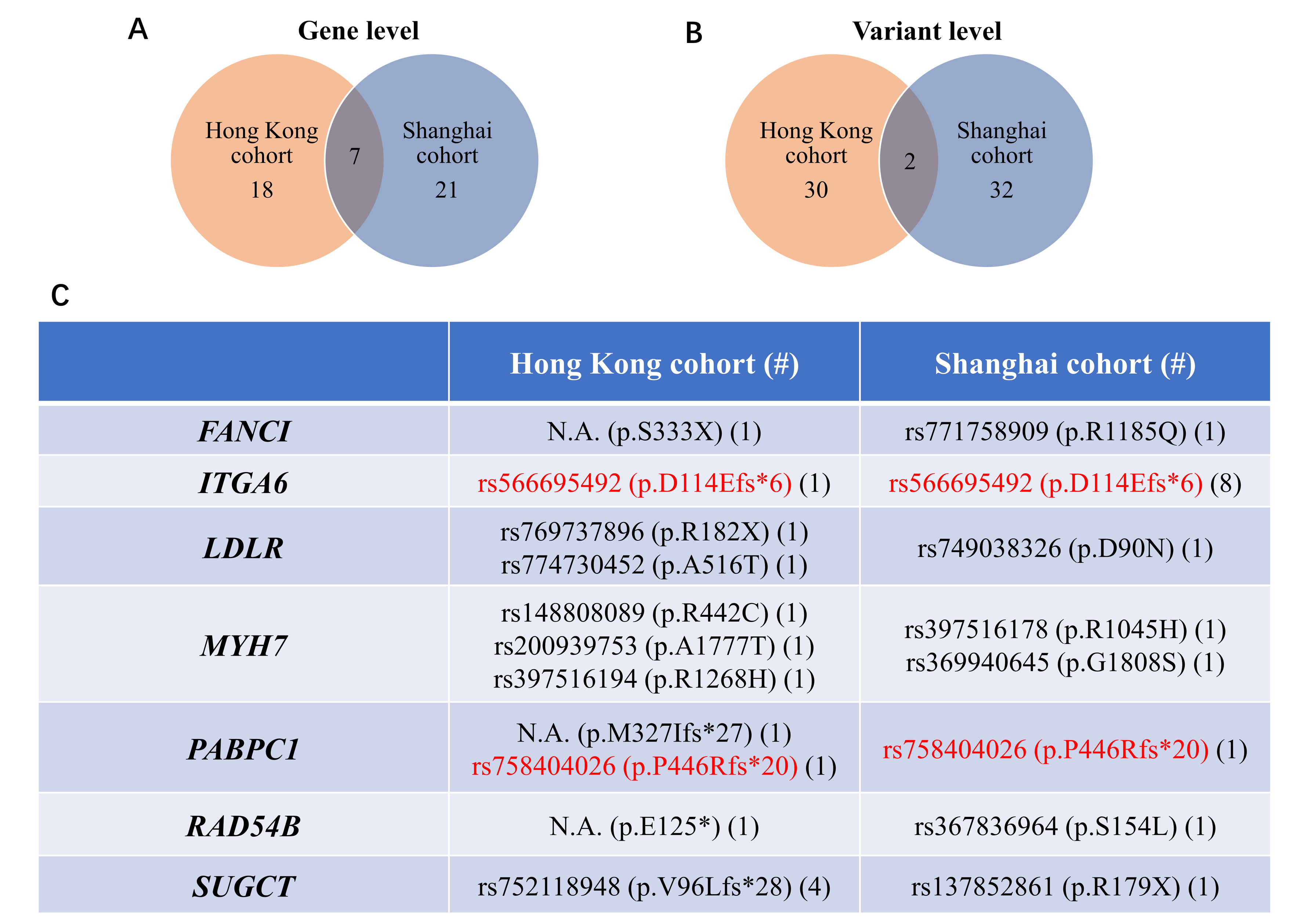

Supplement: S3 Fig — Number of common deleterious (A) genes and (B) variants in both cohorts. (C) Details of common deleterious genes/variants. Common variants are shown in red. The (#) following the variant name represents the number of the variant detected in the cohort. N.A. indicates “not available.” (TIF) [file pgen.1010373.s003.tif]

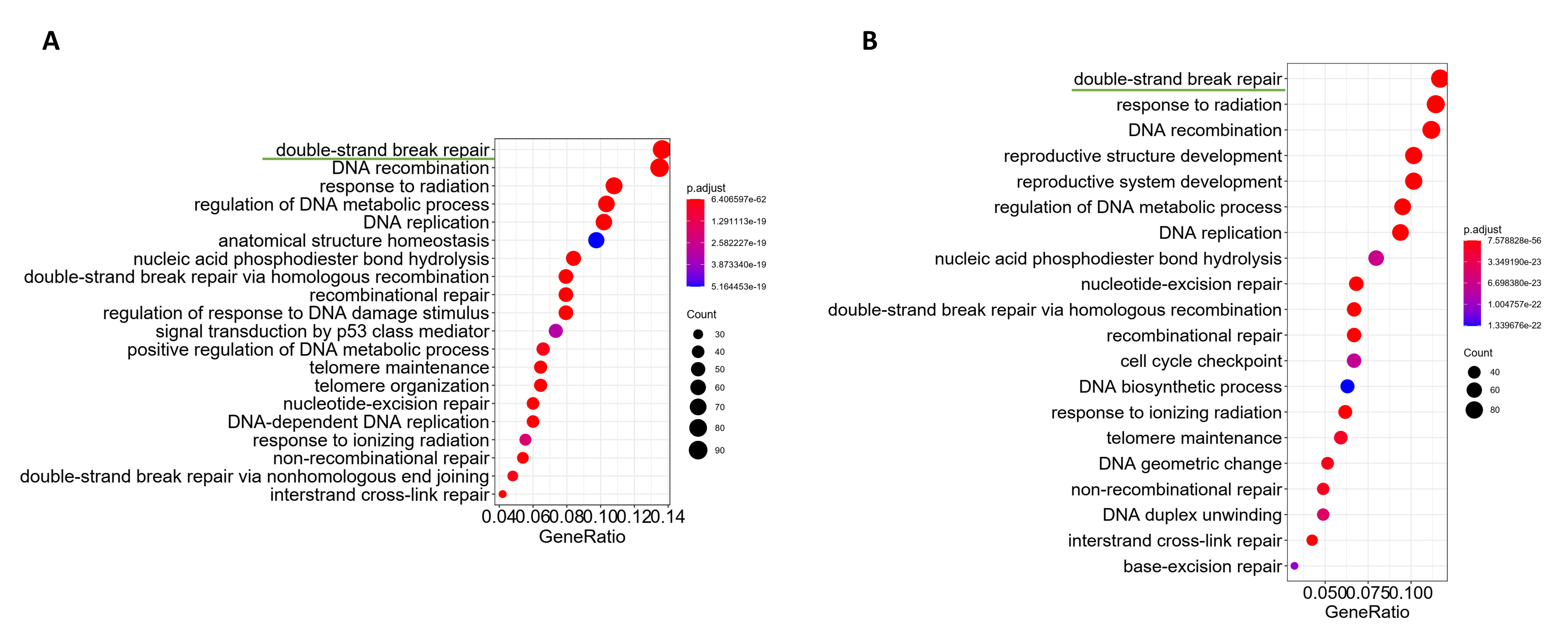

Supplement: S4 Fig — Gene Ontology enrichment analysis of variants of uncertain significance (VUS)-containing genes in the (A) Hong Kong and (B) Shanghai cohorts. The functional categories of GO enrichment were subsequently ranked by the gene ratio (x-axis), which was the percentage of the number of genes present in this GO term over the total number of genes in this category. (TIF) [file pgen.1010373.s004.tif]

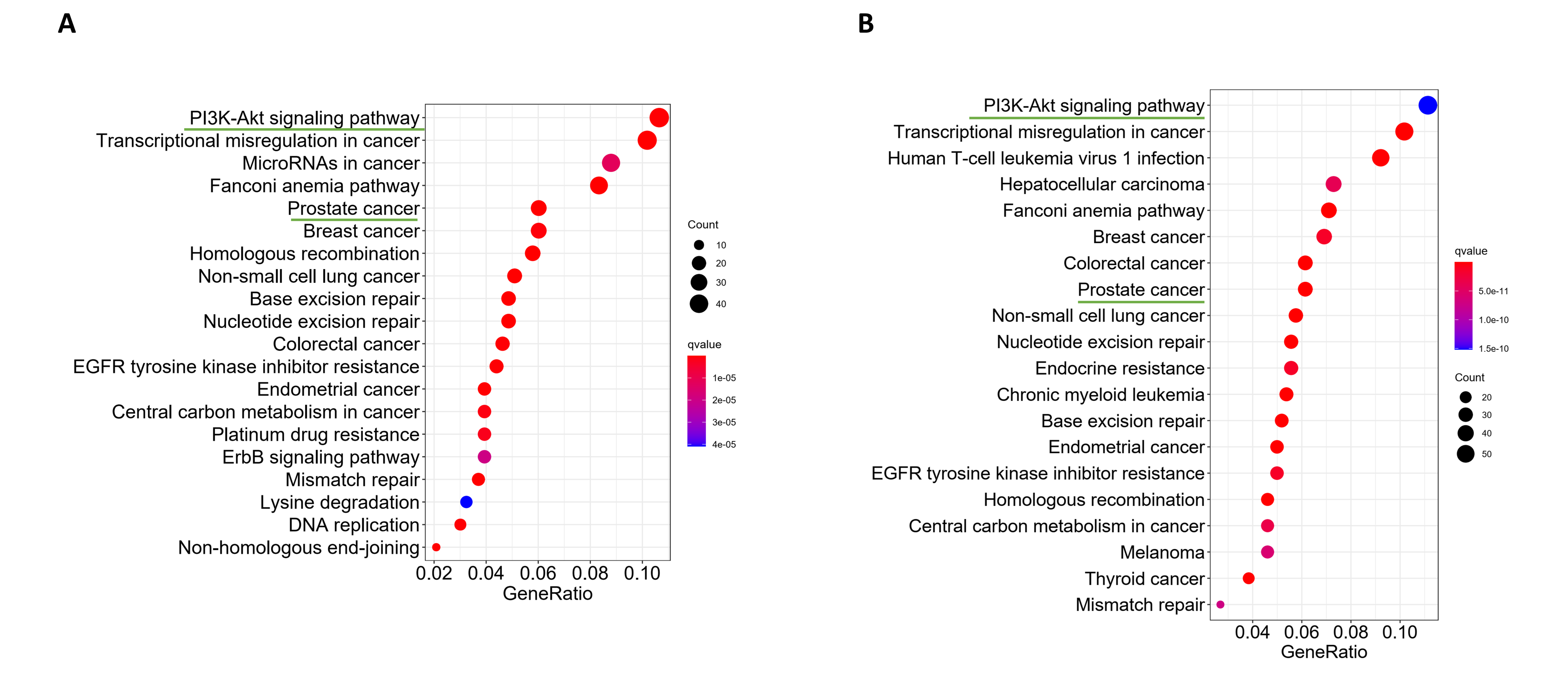

Supplement: S5 Fig — Kyoto Encyclopedia of Genes and Genomes (KEGG) enrichment analysis of variants of uncertain significance (VUS)-containing genes in (A) the Hong Kong and (B) Shanghai cohorts. Top 20 KEGG pathways were ranked by gene ratio (x-axis), which was the percentage of identified genes over the total genes of a given pathway/term. (TIF) [file pgen.1010373.s005.tif]

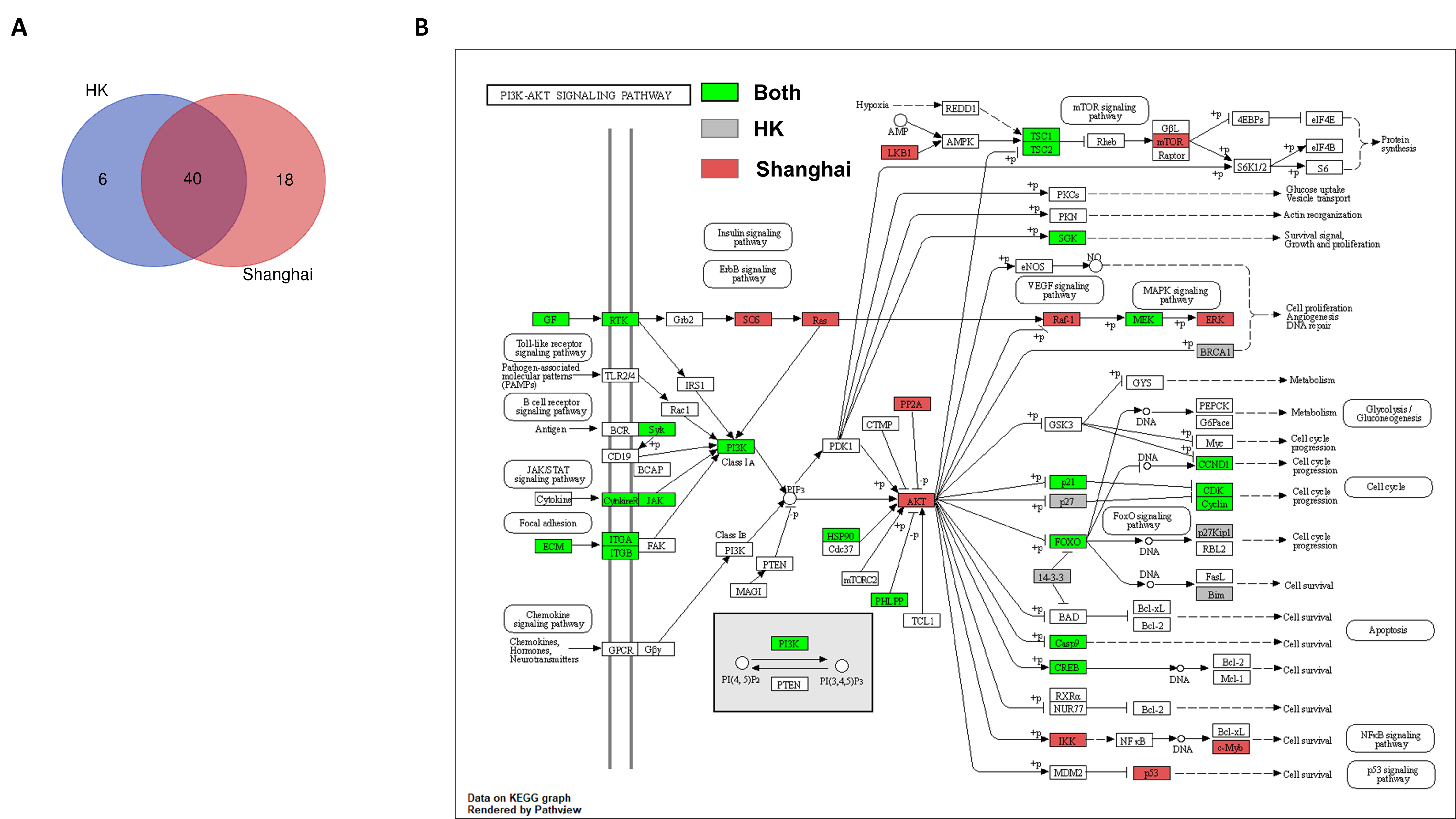

Supplement: S6 Fig — (A) The overlapping VUS-containing genes in PI3K-Akt signaling pathway in two cohorts were summarized in the Venn diagram. (B) VUS-containing genes appeared in both cohorts, in only Hong Kong cohort and in only Shanghai cohort were highlighted in green, gray, and red, respectively. (TIF) [file pgen.1010373.s006.tif]

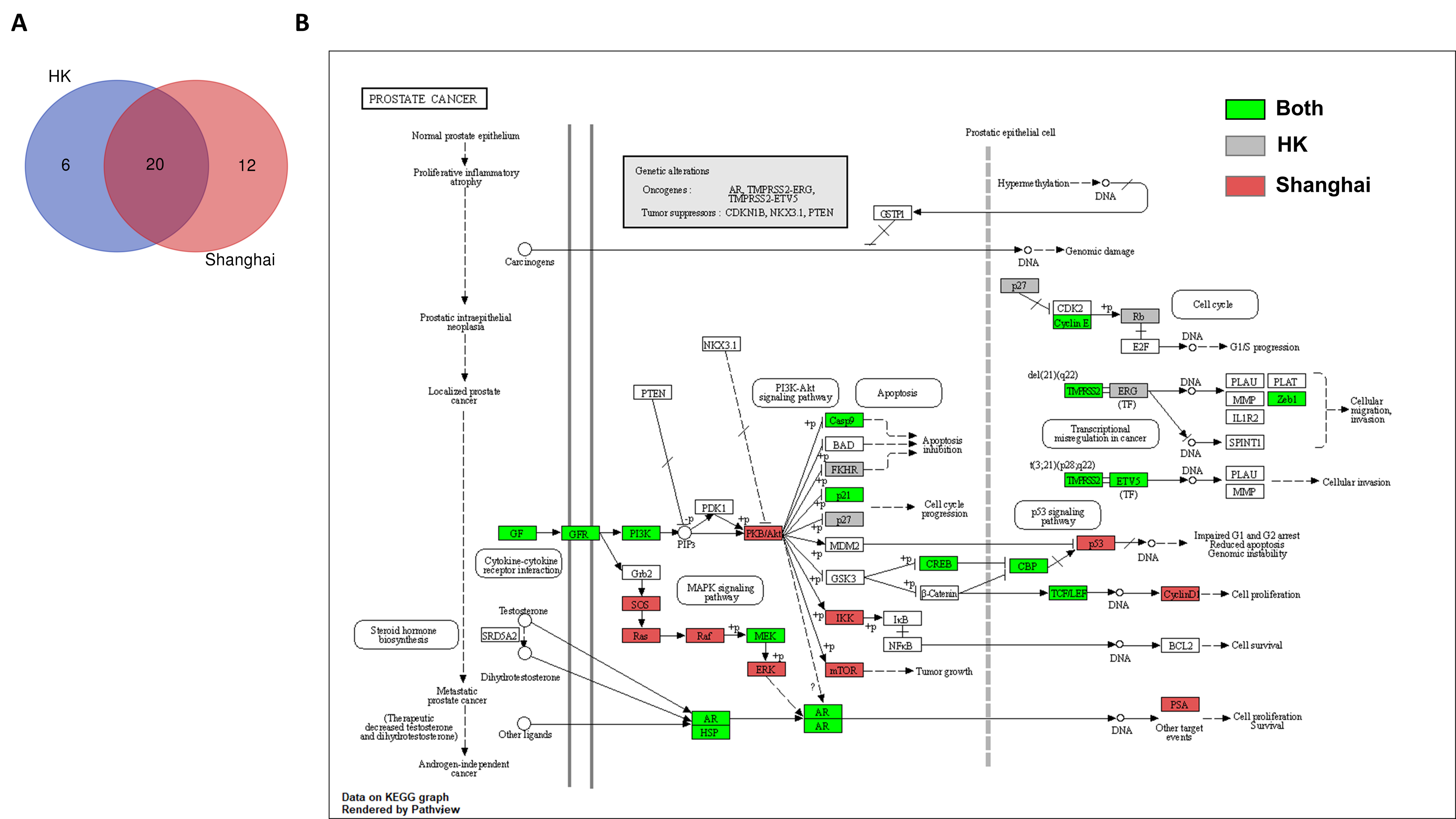

Supplement: S7 Fig — (A) The overlapping VUS-containing genes in prostate cancer pathway in the two cohorts are summarized in the Venn diagram. (B) VUS-containing genes that appeared in both cohorts, in only Hong Kong cohort and in only Shanghai cohort are highlighted in green, gray, and red, respectively. (TIF) [file pgen.1010373.s007.tif]

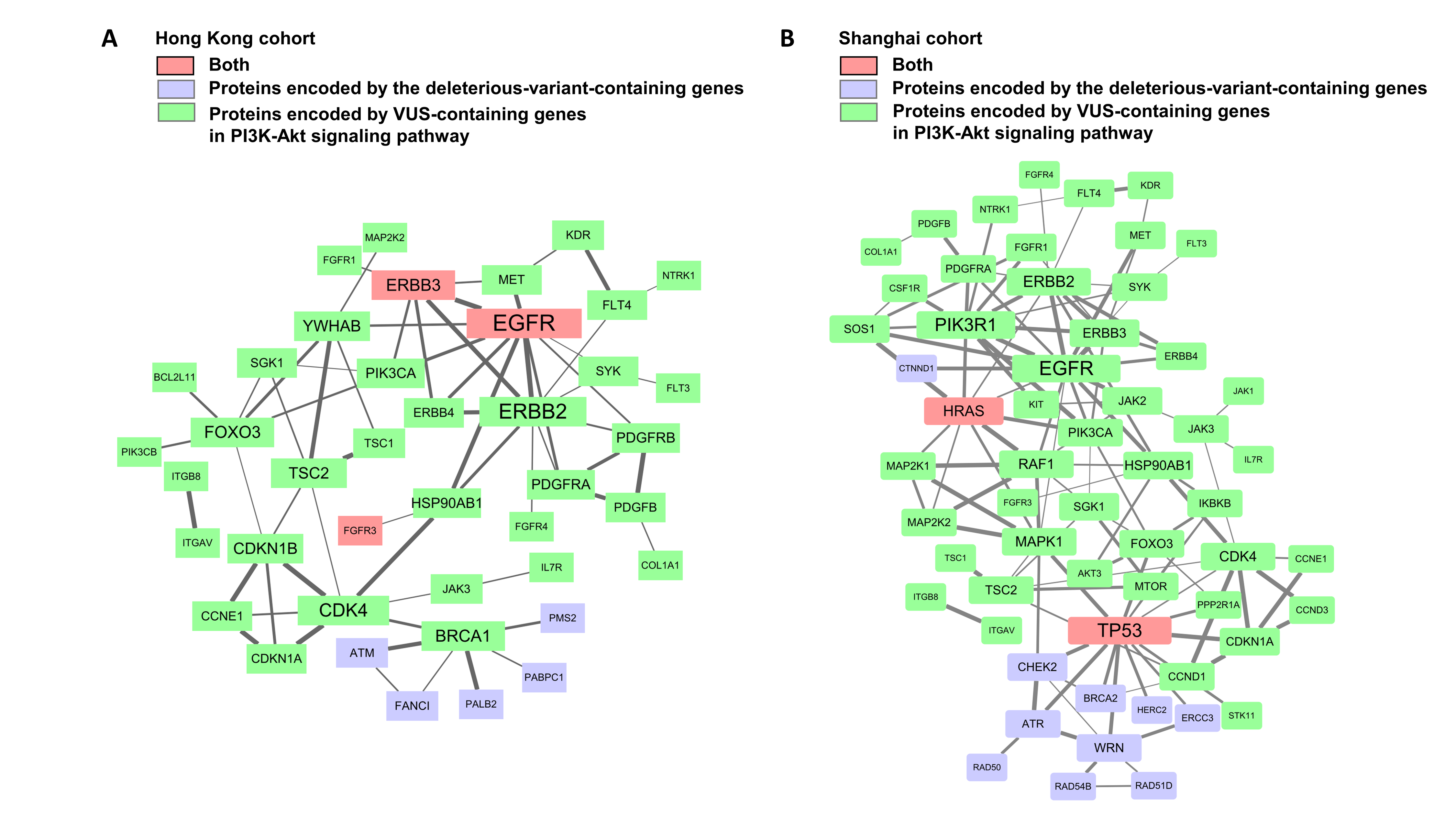

Supplement: S8 Fig — Protein-protein interaction network between proteins encoded by the deleterious-variant-containing genes and the variants of uncertain significance (VUS)-containing genes in the PI3K-Akt signaling pathway in the (A) Hong Kong and (B) Shanghai cohorts. Proteins encoded by both categories, by only deleterious-variant-containing genes, and by only VUS-containing genes in the PI3K-Akt signaling pathway are highlighted in red, blue, and green, respectively. The size of rectangle and font size of protein name are represented depending on the number of edges (connectivity/degree) that each node (protein) has. The more edges, the bigger the node and the font size, the more connective the protein is. The active interaction source is experiments only. The thickness of the edges is represented the strength of data support. The thicker the edges, the more strength the experiments support. (TIF) [file pgen.1010373.s008.tif]

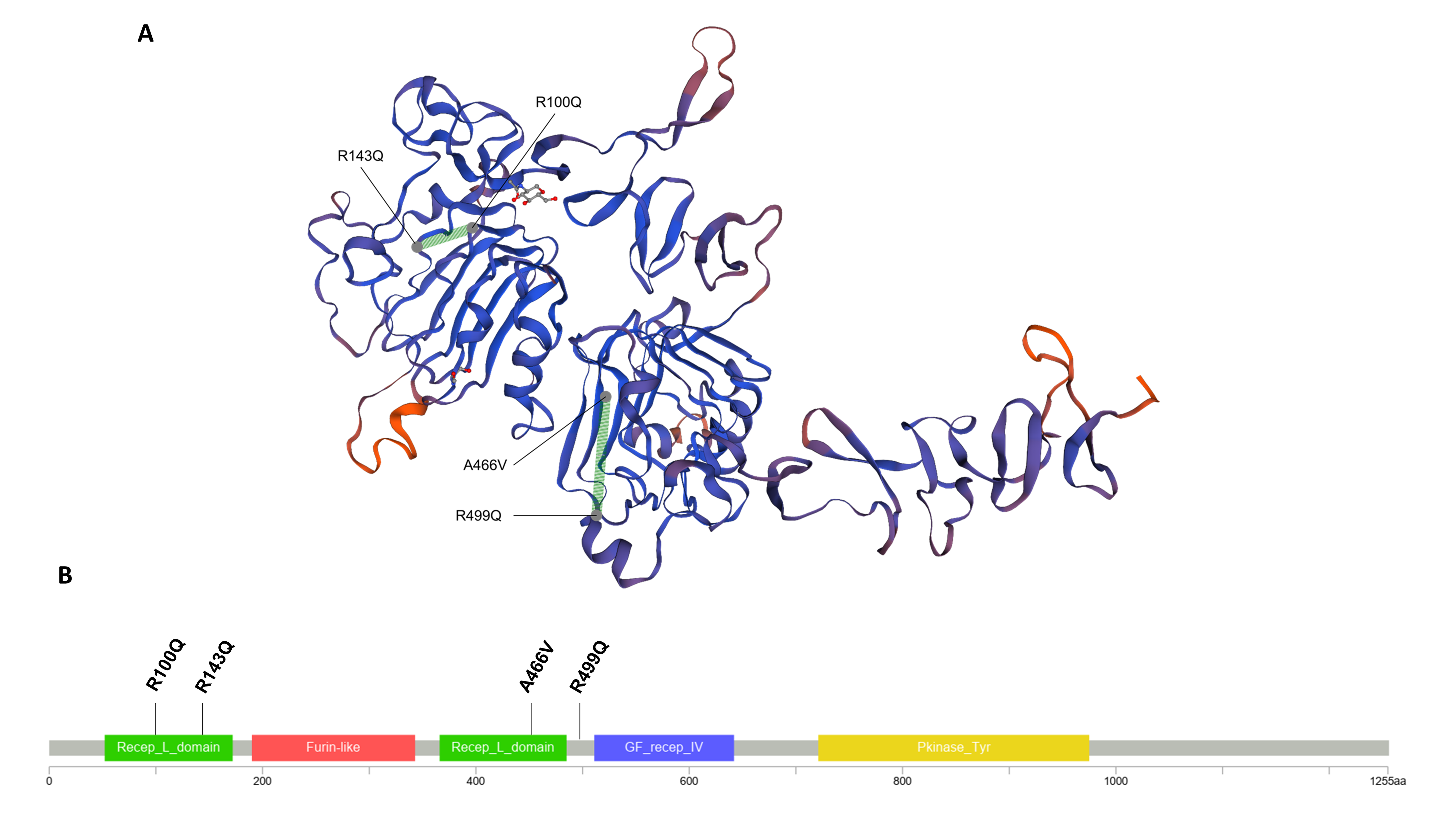

Supplement: S9 Fig — The structure model of ERBB2 (GenBank accession: NM_001289937) was generated using the online tool SWISS-MODEL (https://swissmodel.expasy.org/) with the template of Cryo-EM structure of Receptor tyrosine-protein kinase erbB-2 (SMTL ID: 6bgt.1.A). (A) Four variants in two pairs (closely located within 50 amino acids) were marked and linked, namely R100Q and R143Q (with a molecular distance of 8.31 Å); A466V and R499Q (16.97 Å). (B) The locations of the four variants among the domains of ERBB2. (TIF) [file pgen.1010373.s009.tif]

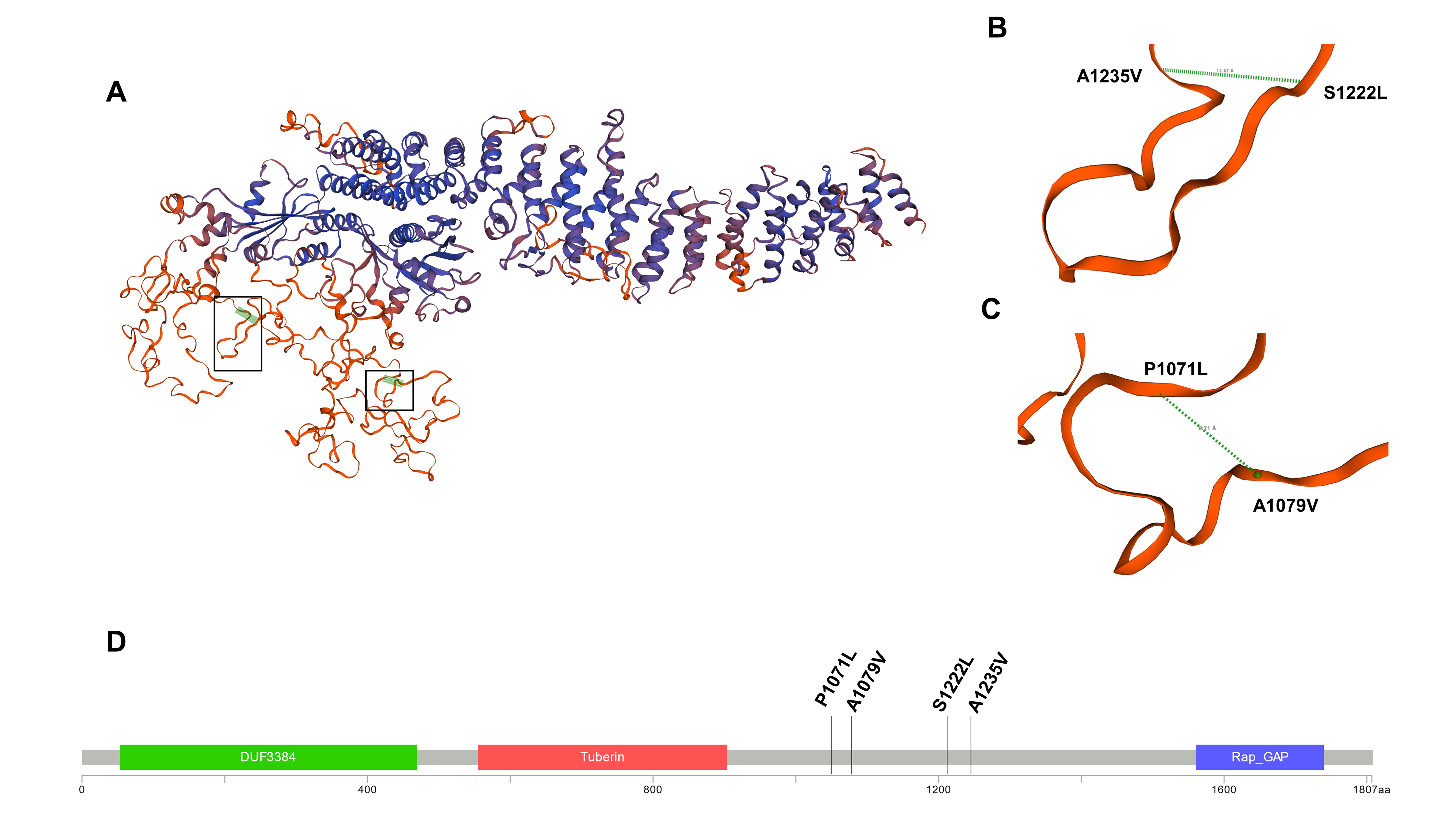

Supplement: S10 Fig — The structure model of TSC2 (GenBank accession: NM_001318831) was generated using the online tool SWISS-MODEL (https://swissmodel.expasy.org/) with the template of Cryo-EM structure of human TSC complex (SMTL ID: 7dl2.1). (A) Four variants in two pairs (closely located within 50 amino acids) were marked and linked, from left to right, A1235V and S1222L, P1071L and A1079V. (B) Structure model of the pair sites A1235V and S1222L (separated by 12.67 Å). (C) Structure model of the pair sites P1071L and A1079V (6.71 Å). (D) Locations of the four variants among the domains of TSC2. (TIF) [file pgen.1010373.s010.tif]

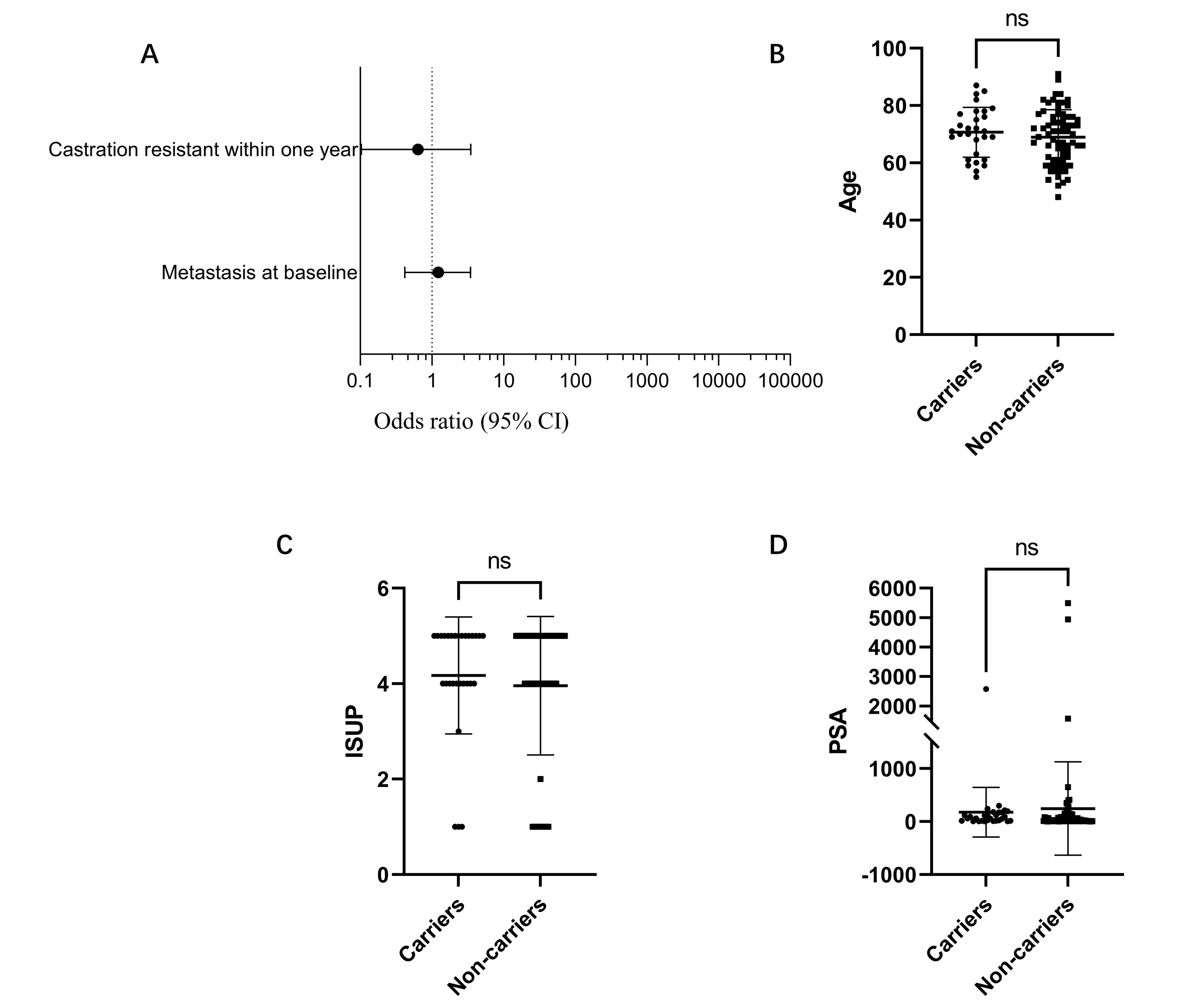

Supplement: S11 Fig — (A) Risk of deleterious-variant-containing genes based on clinical characteristics (metastasis and castration resistance within one year) were calculated. (B, C, and D) The difference of clinical characteristics (age, ISUP grade, and PSA) between the deleterious-variant-containing genes carriers and the non-carriers were analyzed. “ns” indicates “not significant.” (TIF) [file pgen.1010373.s011.tif]

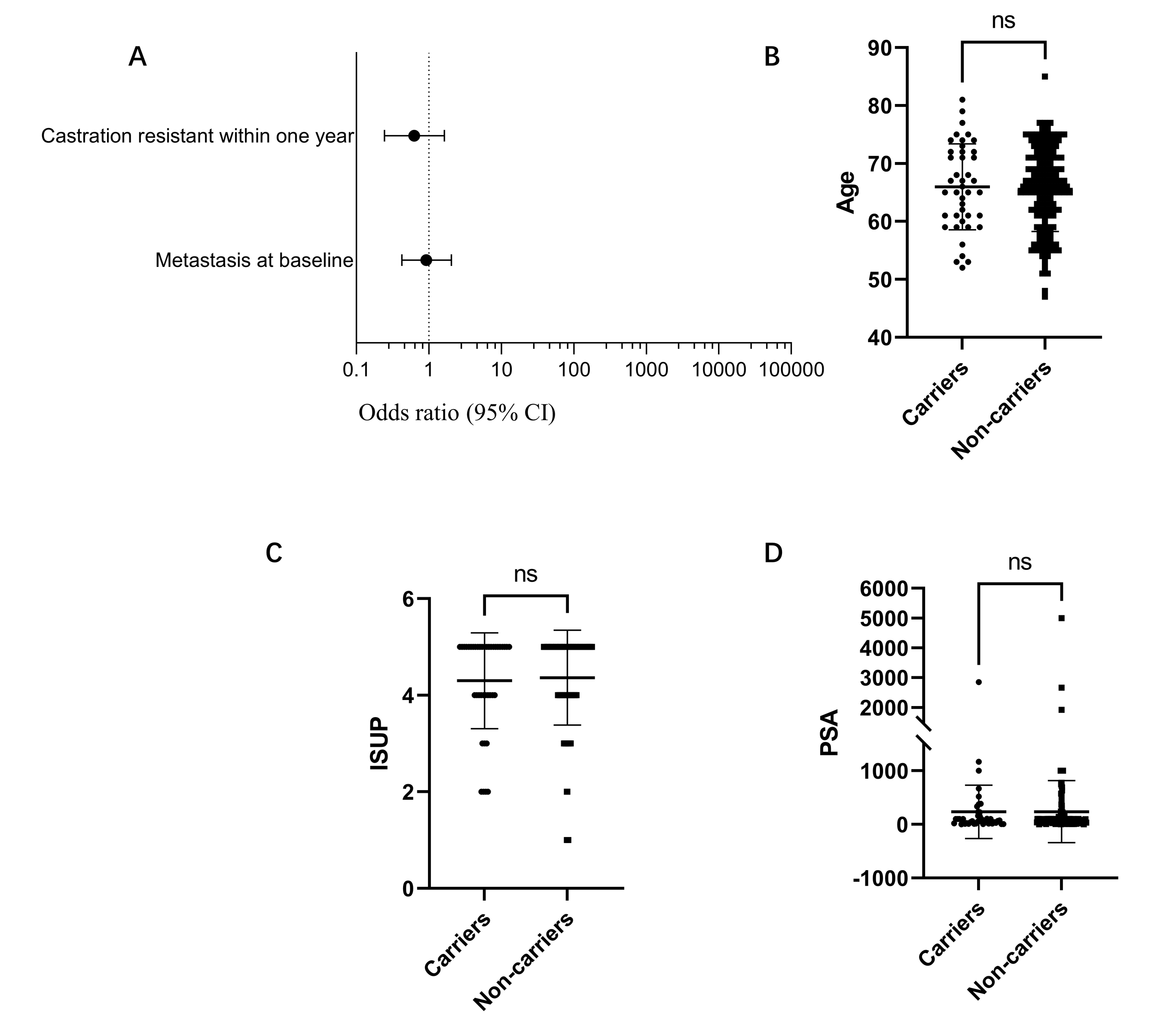

Supplement: S12 Fig — (A) Risk of deleterious-variant-containing based on clinical characteristics (metastasis and castration resistance within one year) were calculated. (B, C, and D) The difference of clinical characteristics (age, ISUP grade, and PSA) between the deleterious-variant-containing genes carriers and the non-carriers were analyzed. “ns” indicates “not significant.” (TIF) [file pgen.1010373.s012.tif]

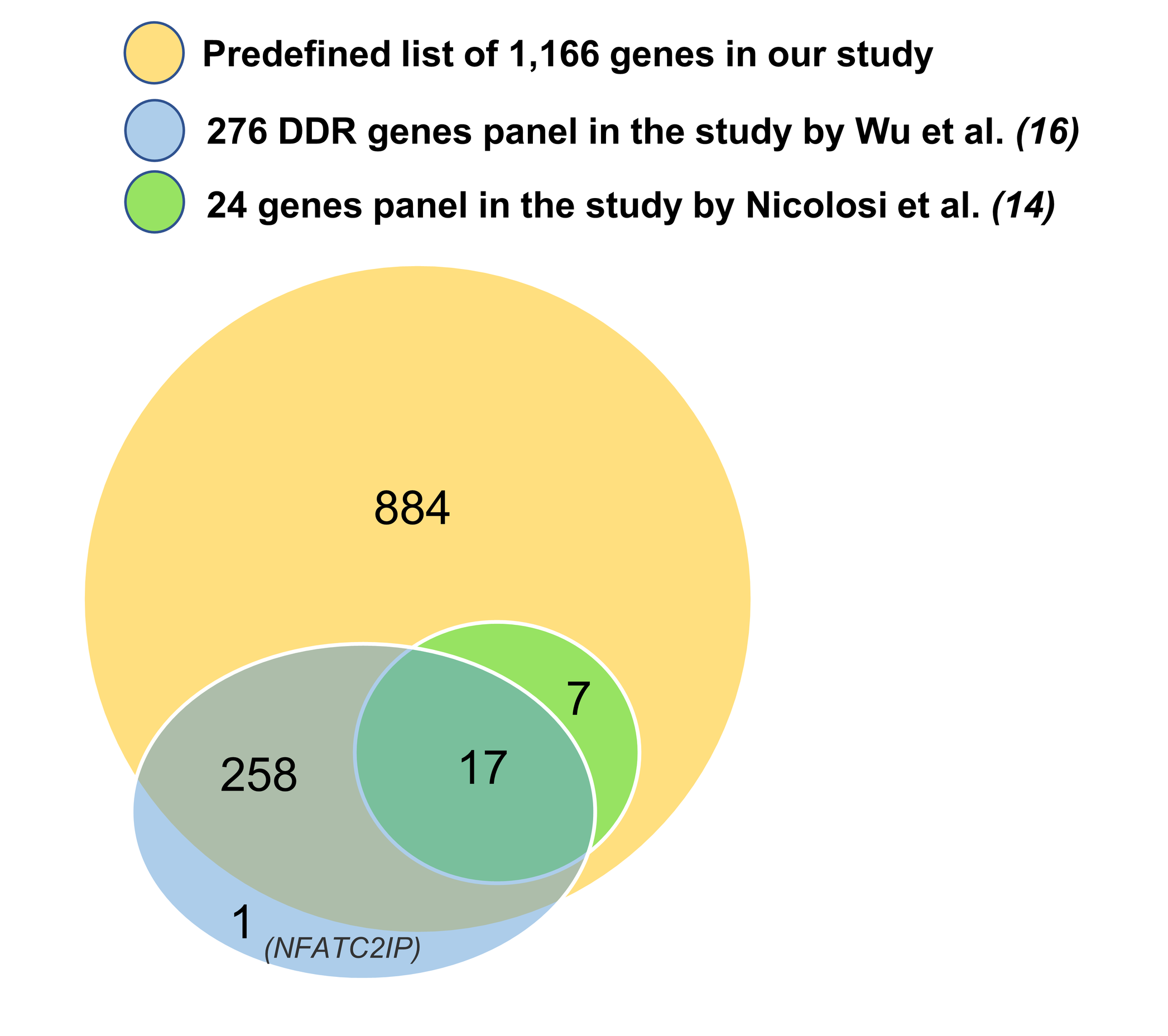

Supplement: S13 Fig — The gene panels from our study, those from the study by Wu et al.[16], and those from the study by Nicolosi et al.[14] are shown in yellow, blue, and green, respectively. The gene names of the gene panels are listed in S10 Table. (TIF) [file pgen.1010373.s013.tif]

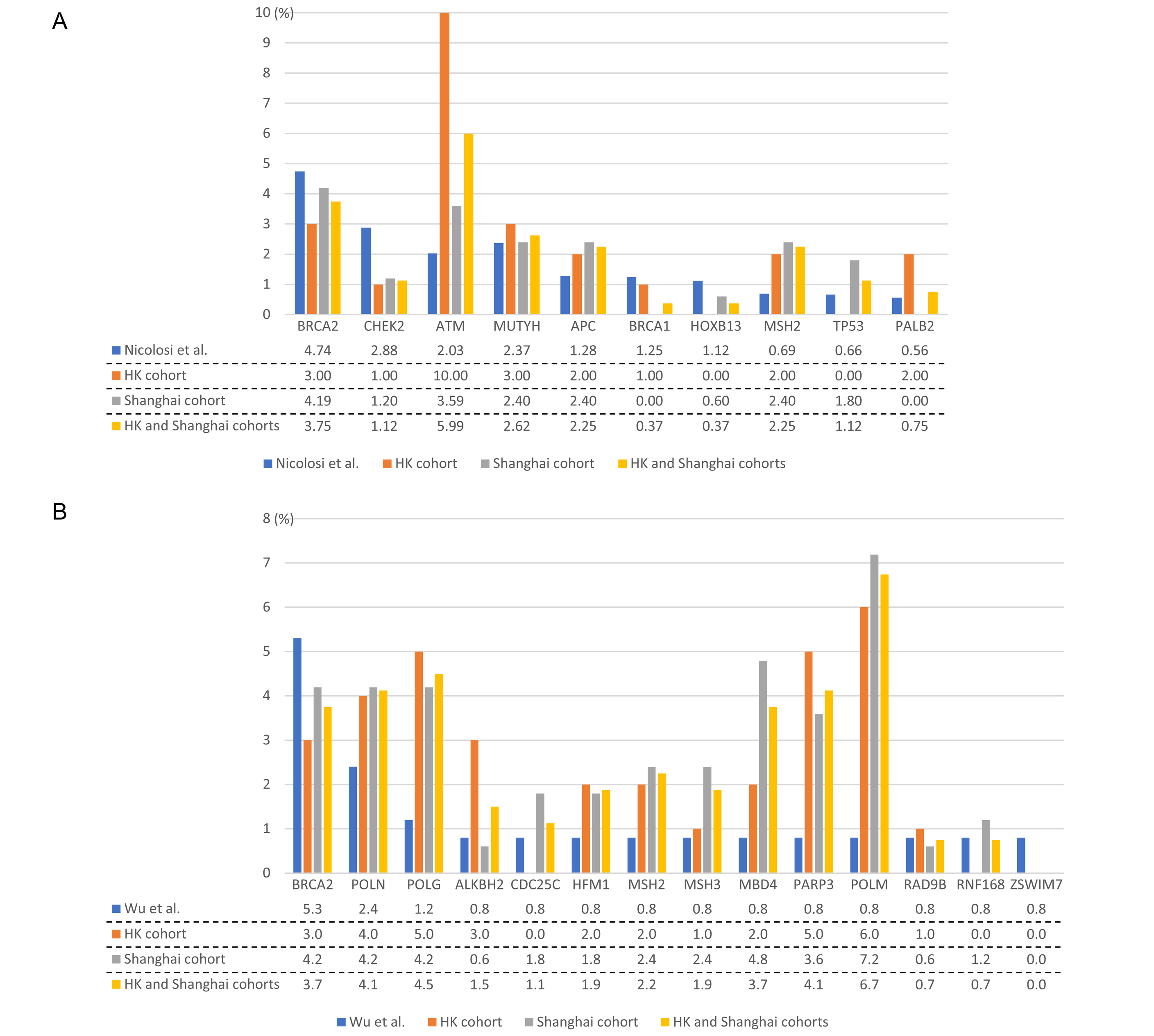

Supplement: S14 Fig — In the Hong Kong and Shanghai cohorts, the mutated genes included those with a deleterious variant and the variant of uncertain significance (VUS)-containing genes. (A) Comparison of the frequency of the top 10 genes from Nicolosi et al.[14] cohort in the Nicolosi et al. cohort, the Hong Kong cohort, the Shanghai cohort, as well as the combined Hong Kong and Shanghai cohort. (B) Comparison of the frequency of the top 10 genes from Wu et al.[16] cohort in the Wu et al. cohort, the Hong Kong cohort, the Shanghai cohort, as well as the combined Hong Kong and Shanghai cohort. (TIF) [file pgen.1010373.s014.tif]

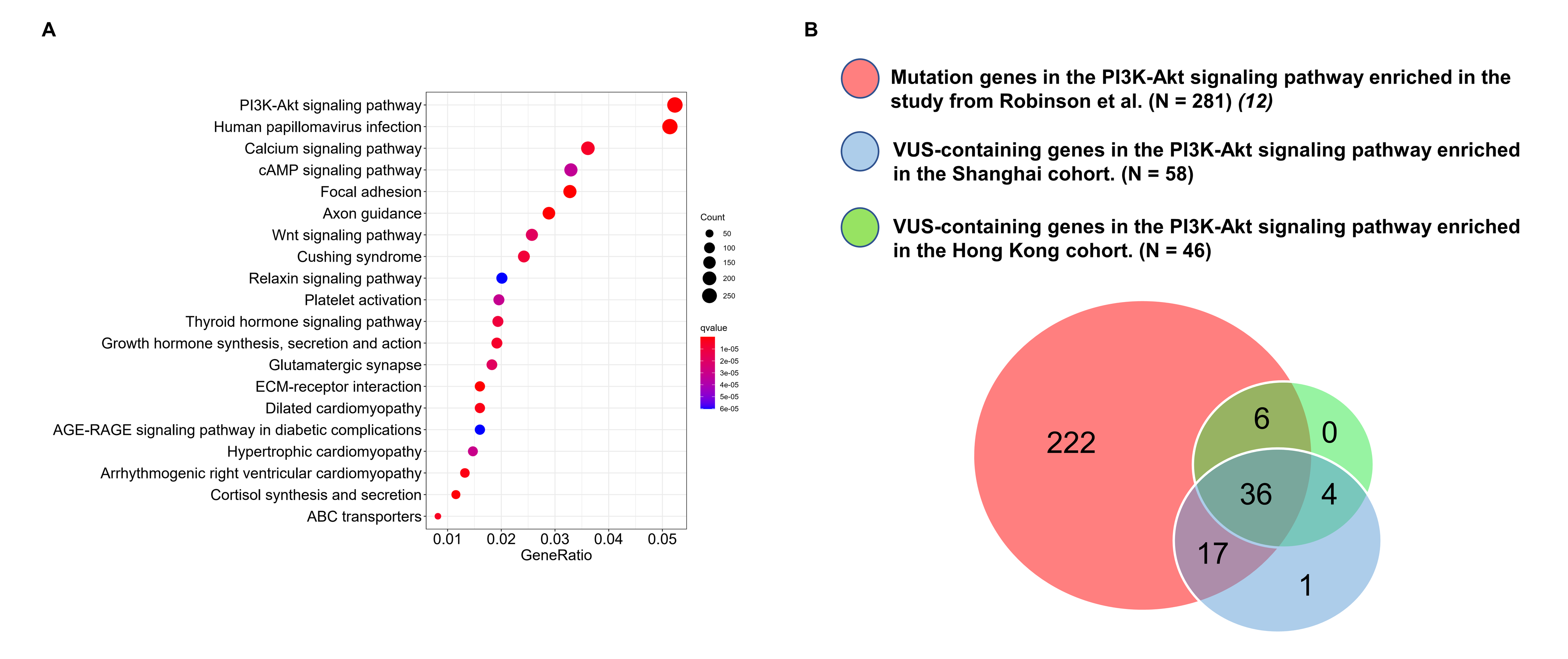

Supplement: S15 Fig — (A) Top 20 KEGG pathways were showed after analyzing 13,972 variant-containing genes (S12 Table) from the study of Robinson et al.[12] (B) Venn diagram of the genes in the PI3K-Akt pathway from the study by Robinson et al.[12] (red), the Hong Kong cohort (green) and the Shanghai cohort (blue). The frequency of the genes in the PI3K-Akt pathway from three cohorts is listed in S13 Table. (TIF) [file pgen.1010373.s015.tif]
